# The long non-coding RNA PIK3CD-AS2 promotes lung adenocarcinoma progression via YBX1-mediated suppression of p53 pathway

**DOI:** 10.1038/s41389-020-0217-0

**Published:** 2020-03-12

**Authors:** Xiufen Zheng, Junying Zhang, Tian Fang, Xiaoxiao Wang, Siwei Wang, Zhifei Ma, Youtao Xu, Chencheng Han, Mengting Sun, Lin Xu, Jie Wang, Rong Yin

**Affiliations:** 1 Department of Thoracic Surgery, Jiangsu Key Laboratory of Molecular and Translational Cancer Research, Jiangsu Cancer Hospital, Jiangsu Institute of Cancer Research, The Affiliated Cancer Hospital of Nanjing Medical University, Nanjing, 210009 China; 2 Department of Pharmacy, The First Affiliated Hospital of Hainan Medical University, Hainan, 570102 China; 3 Clinical Cancer Research Center, Jiangsu Cancer Hospital, Jiangsu Institute of Cancer Research, The Affiliated Cancer Hospital of Nanjing Medical University, Nanjing, 210009 China; 4 Department of Comparative Medicine, Jinling Hospital, Clinical School of Medical College of Nanjing University, Nanjing, 210093 China; 5 GCP Research Center, Affiliated Hospital of Nanjing University of Chinese Medicine, Jiangsu Province Hospital of TCM, Nanjing, 210029 China; 6 Department of Tumor Biobank, Jiangsu Cancer Hospital, Jiangsu Institute of Cancer Research, The Affiliated Cancer Hospital of Nanjing Medical University, Nanjing, 210009 China; 7 Department of Science & Technology, Jiangsu Cancer Hospital, Jiangsu Institute of Cancer Research, The Affiliated Cancer Hospital of Nanjing Medical University, Nanjing, 210009 China

**Keywords:** Non-small-cell lung cancer, Oncogenes

## Abstract

The underlying mechanisms of long non-coding RNAs (lncRNA) participating in the progression of lung cancers are largely unknown. We found a novel lncRNA, PIK3CD antisense RNA 2 (PIK3CD-AS2), that contributes to lung adenocarcinoma (LUAD) progression. The expression characteristics of PIK3CD-AS2 in LUAD were analyzed using microarray expression profile, The Cancer Genome Atlas (TCGA) and Gene Expression Omnibus (GEO) datasets, and validated in 92 paired LUAD tissues by chromogenic in situ hybridization. Our data confirmed that PIK3CD-AS2 expression is a crucial regulator of LUAD progression and associated with shorter patient survival. In vitro studies showed that PIK3CD-AS2 increased cell growth and slowed apoptosis in p53^wt^ cells but not in p53^null^ cells. Mechanically, it is demonstrated that PIK3CD-AS2 bound to and maintained the stability of Y-box binding protein 1 (YBX1), a potent destabilizer of p53, by impeding its ubiquitination and degradation. Downexpression of YBX1 reversed PIK3CD-AS2-mediated inhibition of p53 signaling. Additionally, the therapeutic effect evaluation of a locked nuclear acid (LNA) specifically targeting PIK3CD-AS2 showed an anti-tumor activity in mice with A549 cells xenograft and p53 wild-type LUAD patient-derived tumor xenograft (PDTX) model. Clinically, the high expression of PIK3CD-AS2 showed a poor disease-free survival in p53 wild-type patients in TCGA database. Our findings suggest that PIK3CD-AS2 regulates LUAD progression and elucidate a new PIK3CD-AS2/YBX1/p53 signaling axis, providing a potential lncRNA-directed therapeutic strategy especially in p53 wild-type LUAD patients.

## Introduction

Lung cancer, predominantly non-small cell lung cancer (NSCLC) is the deadliest malignancy worldwide, accounting for the largest number of cancer-related mortality^1^. Among NSCLC, lung adenocarcinoma (LUAD) is the most common histopathologic type. Patients with early stage of LUAD usually have better outcomes but only 23% of lung cancer cases were found at an early stage. Once the cancer has spread, it is often shown a limited survival.

Targeted therapy based on driver genes opened a new era of cancer treatment in the past decades. Data from omics profiling studies have identified lots of driver gene mutations. It is well-known that the presence of epidermal growth factor receptor (EGFR)-activating mutation makes at least 20% of LUAD patients eligible to tyrosine kinase inhibitor (TKI) therapy^2,3^. Anaplastic lymphoma kinase (ALK) rearrangement are found in 7–9% of LUAD cases, and crizotinib has been demonstrated to increase patient median progression-free survival to 8–10 months^4^. During types of NSCLC driver mutations, genetic abnormalities in the tumor protein 53 (TP53) gene are the most frequent alterations, attracting plenty of therapeutic studies^5,6^. Two p53 reactivating compounds, APR-246 and COTI-2, have been found to display anticancer activity in phase I clinical trials^7^. Given the wild-type form of p53 is dominant over the mutant, novel treatments aiming to sustain wild-type p53 activity become an alternative opportunity. Growing evidences suggest that restoring wild-type p53 function by gene-replacement strategies could suppress lung cancer progression^8–10^, indicating a potential clinical applicability, although these p53 restoration approaches still remain preliminary.

Non-coding RNAs (ncRNAs) are new emerging targets for tumor therapy. They have been identified to function in cancer as oncogenic drivers and tumor suppressors, or as key regulators which form an orchestrated interaction modulating well-known genes^11^. In NSCLC, it is demonstrated that estrogen receptor β can promote cancer vasculogenic mimicry formation and cell invasion via altering long non-coding RNA (lncRNA) MALAT1/miR145–5p/NEDD9 signaling^12^. Environmental carcinogen nickel exposure led to lncRNA MEG3 downregulation, further initiated PHLLPP1 transcriptional decrease and hypoxia-inducible factor-1α protein translational increase, eventually inducing tumor malignant progression^13^. Our group focuses on ncRNAs in NSCLC and previously identified a novel lncRNA LUADT1 promoting cell cycle and tumor growth via epigenetically blocking p27 expression^14^. We also demonstrated high expressed SBF2-AS1 functioning as a competitive endogenous RNA facilitates the progression of lung cancer^15^. In addition, we first found that a proto-oncogenic circular RNA (circPRKCI) coming from one of the most frequent genomic amplificated regions 3q26.2, that is significantly overexpressed in LUAD tissues and promotes tumorigenesis^16^.

Here, we investigated another LUAD-upregulated lncRNA PIK3CD antisense RNA 2 (PIK3CD-AS2), previously reported in our lncRNA microarray GSE66654^14^, as a candidate oncogene contributed to cancer growth via suppressing p53 signaling. Lack of PIK3CD-AS2 restored p53 expression levels and offered a possibility of anti-tumor activity. Our study provides the first evidence of the biological function of lncRNA PIK3CD-AS2 and its underlying role in YBX1/p53 axis, which could be used as a potential therapeutic target for lung cancer clinical practice.

## Results

### PIK3CD-AS2 is overexpressed in LUAD and associated with tumor size and poor survival

**Fig. 1:**
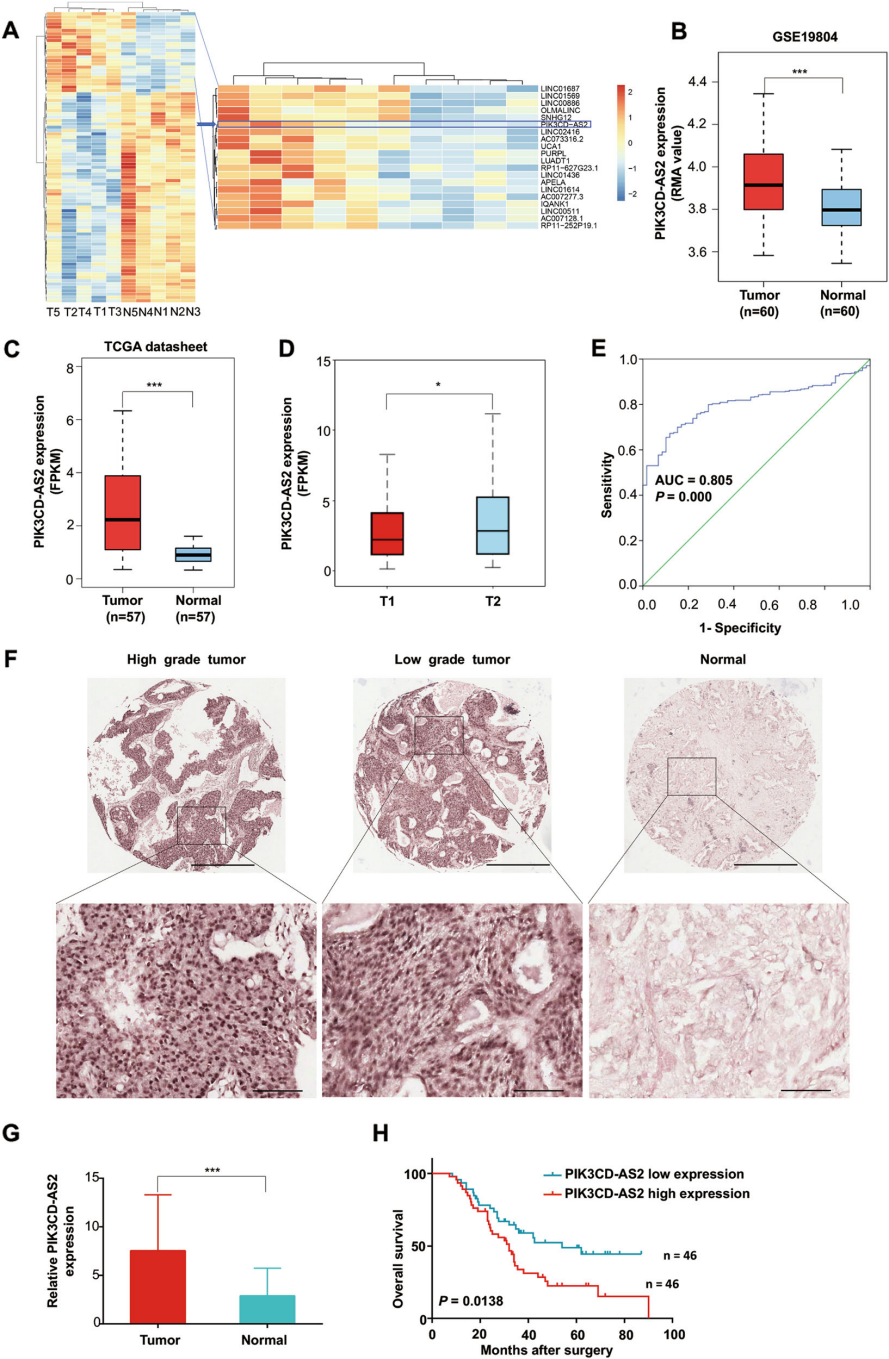
PIK3CD-AS2 is overexpressed in lung adenocarcinoma cancer (LUAD) and associated with tumor size, histological grade and poor survival. **a** Total RNA harvested from human LUAD tumor tissues and matched non-tumor tissues was screened by microarray analysis (fold-change ≥ 2; *P* ≤ 0.01; *n* = 5). Left: the cluster heat map classified as differentially expressed lncRNAs. Right: 20 top-ranked upregulated lncRNAs were shown in LUAD tumors. Upregulated (Red) and downregulated (Blue) lncRNAs in LUAD. **b**, **c** PIK3CD-AS2 expression in LUAD and normal lung tissues from **b** GEO data sets GSE19804 normalized using the robust multichip averaging (RMA) values and **c** TCGA transcriptome profiling represented as FPKM, fragments per kilobase of exon per million fragments mapped reads. Data are presented as median ± 1.5 IQR. *P* values were determined by *t*-test. ***P < 0.001 (GEO: *n* = 60; TCGA: *n* = 57). **d** PIK3CD-AS2 expression of early-stage (I–IIa) lung cancer patients in TCGA data was separated by tumor size, and expressed as median ± 1.5 IQR. *P* value was determined using unpaired *t*-test. *P < 0.05. **e** Receiver-operating characteristic (ROC) curve calculated sensitivity and specificity of PIK3CD-AS2 expression level for prediction of LUAD (tumor, *n* = 513, normal, *n* = 59, area under ROC curve = 0.805, *P* = 0.000). **f** Representative images of in situ hybridization (ISH) of PIK3CD-AS2 in the TMA of 92 LUAD patients samples. Scale bars: 500 μm and 100 μm (insets). **g** PIK3CD-AS2 expression measured by ISH was scored by pathologists blind to the study design. Data are presented as mean ± SD. *P* value was determined by paired *t*-test. ***P < 0.001. **h** Kaplan–Meier overall survival (OS) analysis of high PIK3CD-AS2 expression and low PIK3CD-AS2 expression in LUAD patients (log rank test, *P* = 0.0138, *n* = 92).

By analyzing mRNA and lncRNA expression profiles from our previous LUAD microarray data (GSE66654), we found that PIK3CD-AS2 had more than 13-fold changes in tumor tissues vs. paired non-tumor tissues (Fig. 1a). It was consistent with the results of both GSE19804 cohort, including 60 paired LUAD samples (*P* < 0.001, Fig. 1b) and the 57 paired LUAD samples extracted from TCGA (*P* < 0.001, Fig. 1c). The high transcription level of PIK3CD-AS2 was associated with tumor size in early-stage (I–IIa) lung cancer (*P* < 0.05, Fig. 1d). Accordingly, the area under the receiver operating characteristic (ROC) curve analyzing the sensitivity and specificity of PIK3CD-AS2 expression in LUAD was 0.805 (95% CI = 0.765–0.845; *P* < 0.001; Fig. 1e), indicating PIK3CD-AS2 could be a new predictive marker.

We further evaluated the PIK3CD-AS2 expression profiles in our clinical LUAD tissues (92 paired paraffin-embedded tissues) by chromogenic in situ hybridization (CISH), and observed a similar expression tendency (Fig. 1f, g). As shown in Supplementary Table S1, greater PIK3CD-AS2 expression was positively associated with tumor size (*P* = 0.03) and histological differentiation (*P* = 0.04). Kaplan–Meier analysis and log-rank test showed a remarkable correlation between tumor-associated PIK3CD-AS2 expression and worse overall survival outcome (*P* = 0.0138; Fig. 1h). Furthermore, we found PIK3CD-AS2 is also upregulated in several solid tumors such as lung squamous, liver, and kidney renal papillary cell carcinoma (KIRC) (Supplementary Fig. S1), suggesting that upregulation of PIK3CD-AS2 might be general. These data concluded that PIK3CD-AS2 plays a carcinogenic role regarding LUAD.

### PIK3CD-AS2 promotes LUAD cells malignant behavior in vitro

**Fig. 2:**
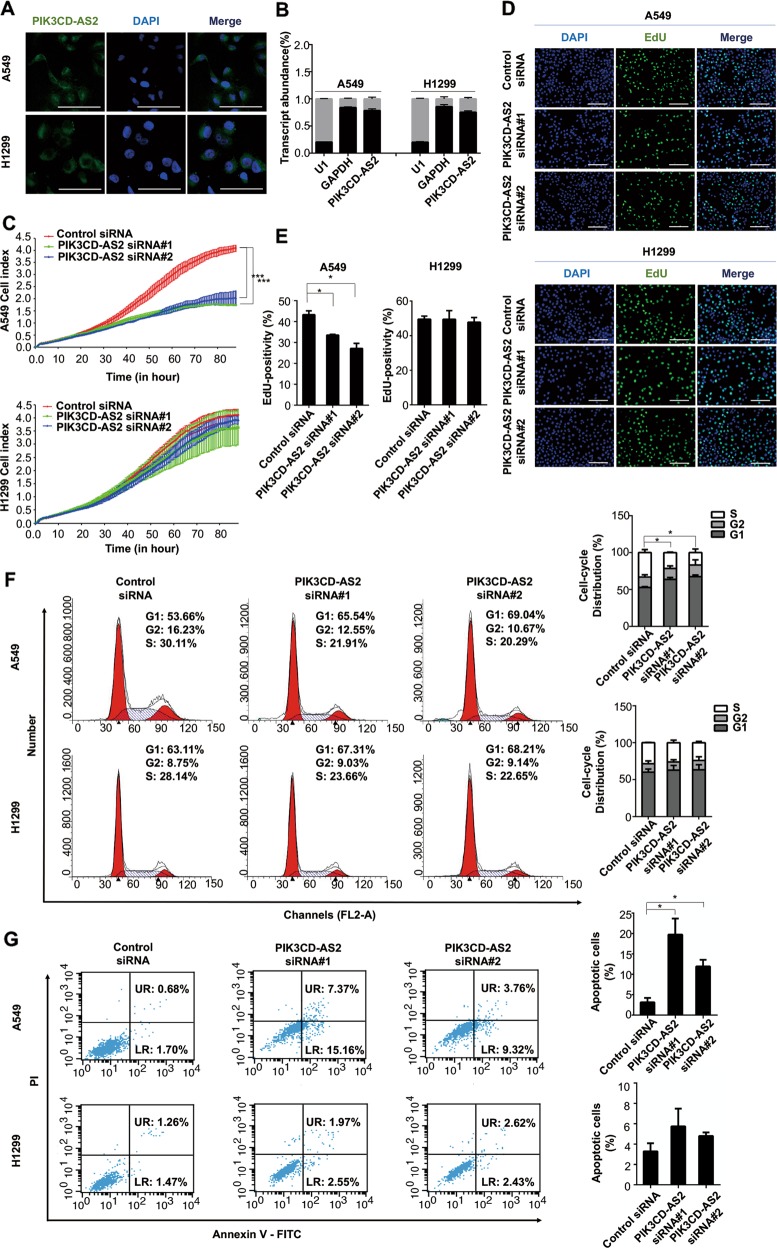
Depletion of PIK3CD-AS2 abrogates LUAD cell progression in vitro. **a** Localization of PIK3CD-AS2 was evaluated by RNA ISH in A549 and H1299 cells. RNA ISH was performed with Dig-labeled probes (green) and nuclei were labeled with DAPI (blue). Scale bars: 50 μm. **b** Transcript levels of PIK3CD-AS2, U1 (nuclear marker) and GAPDH (cytoplasmic marker) in the nuclear and cytoplasmic fractions obtained from A549 and H1299 cells were quantified by qRT-PCR. **c**–**g** A549 and H1299 cells were transfected with PIK3CD-AS2 siRNA or control siRNA, respectively. Twenty-four hours after transfection, 5 × 10^3^ cells per well were seeded in an E-plate and monitored continuously for 90 h using the xCelligence real-time cell analyzer (RTCA; **c**). ***P < 0.001 determined by unpaired *t*-test. Forty-eight hours after transfection, some cells were evaluated by an EdU incorporation assay (**d**, **e**). Representative images show proliferation of A549 and H1299 cells labeled with EdU (green) and nuclei stained with DAPI (blue). Scale bars: 50 μm. The proportion of EdU-positive cells in A549 and H1299 cells was quantified. Values represent mean ± SD of three independent experiments. Statistical analysis was carried out using unpaired *t* test. *P < 0.05; ***P < 0.001. Meanwhile, the effect of PIK3CD-AS2 on phases of cell cycle (**f**) and apoptosis (**g**) in A549 and H1299 cells was measured by flow cytometry. Left: representative plots showing the cell cycle distribution or cell apoptosis. Right: percentages of G_0_, G_1_, and S phage, or apoptotic cells were calculated, respectively. Values represent mean ± SD of three independent experiments. Statistical analysis was performed using unpaired *t*-test. *P < 0.05.

PIK3CD-AS2 has three transcripts, in which ENSG00000231789 is the longest one with three exons. qRT-PCR data demonstrated ENSG00000231789 contributed to nearly all upregulation of PIK3CD-AS2 in LUAD tissues (Supplementary Fig. S2). Analysis of the sequences by CPAT, ORF finder and CPC2 online software suggested a low protein coding potentiality of PIK3CD-AS2 indicating which plays a role at RNA level (Supplementary Fig. S3). To verify the biological function of PIK3CD-AS2, we firstly examined the baseline level of PIK3CD-AS2 in six LUAD cell lines and a normal human bronchial epithelial cell line (HBE1). We found PIK3CD-AS2 is generally up-regulated in LUAD cell lines compared to HBE1 cells. Then we selected PIK3CD-AS2 highly expressed H1299 and A549 cells for following experiments (Supplementary Fig. S4a). By RNA fluorescence in situ hybridization (FISH) and cell fractionation analysis, we confirmed PIK3CD-AS2 is mainly expressed in the cytoplasm of H1299 and A549 cells, whereas less in the nucleus (Fig. 2a, b).

We next knocked down PIK3CD-AS2 using two different high-efficiency siRNAs (Supplementary Fig. S4b). We found that silencing PIK3CD-AS2 decreased the proliferation of A549, but not of H1299 cells (*P* < 0.001 vs. control; Fig. 2c–e). The downregulation of PIK3CD-AS2 also induced cell cycle arrest at G0/G1 phase and apoptosis in A549 cells (Fig. 2f, g). However, we failed to observe a similar alteration in p53^null^ H1299 cells. In addition, si-PIK3CD-AS2 inhibited cell invasion and migration, especially in A549 cells (Supplementary Fig. S4c, d). These data suggested that the proto-oncogenic role of PIK3CD-AS2 in LUAD might be dependent on p53 status.

### PIK3CD-AS2 induces tumor progression of LUAD cells in vivo

**Fig. 3:**
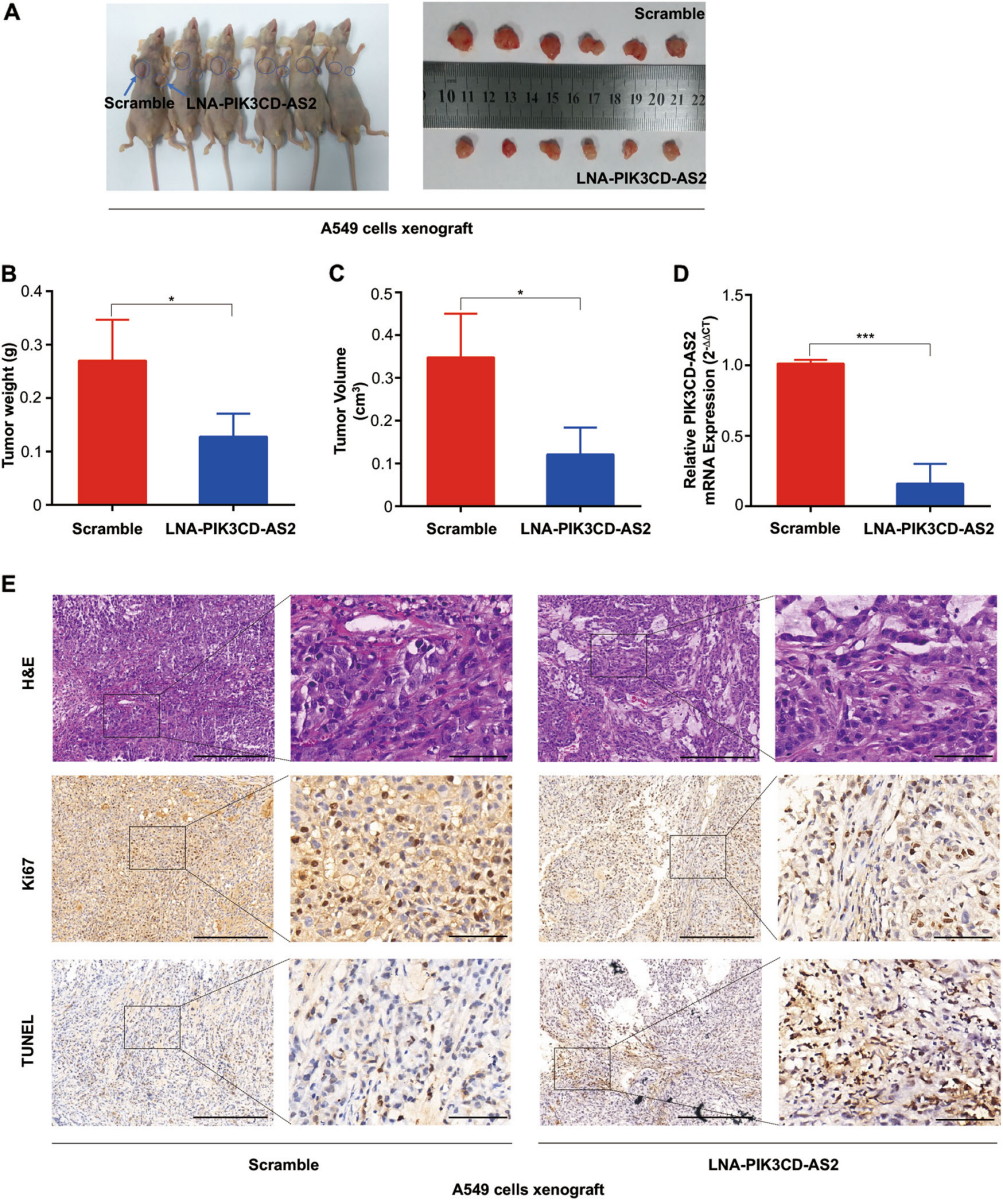
Loss of PIK3CD-AS2 represses tumorigenesis of LUAD cells in vivo. A total of 5 × 10^6^ A549 cells were injected subcutaneously into the armpits of NOD/SCID mice and LNA-PIK3CD-AS2 (left armpit) or scramble control (right armpit) at 1.5 mg/kg was administrated by intratumoral injection twice-weekly for the first week and then once per week for the next 5 weeks. The mice were killed and analyzed 6 weeks after A549 cells xenograft. **a** Representative images of mice (left) and xenograft tumors (right) after LNA treatment were shown. *n* = 6. **b**, **c** Tumor weights (**b**), and tumor volume (**c**) was quantitative analyzed. **d** PIK3CD-AS2 expression in xenograft tumors was measured by qRT-PCR. **e** Representative images of H&E staining, IHC staining with anti-Ki67 and TUNEL assay labeling of mice xenograft tumor sections were shown. Scale bars: 500 and 100 μm (insets). Data are expressed as means ± SD (**b**–**d**). Statistical analysis was carried out using unpaired *t*-test. *P < 0.05; ***P < 0.001.

To investigate the effect of PIK3CD-AS2 on tumorigenesis in vivo, we used Locked Nucleic Acid (LNA^TM^) oligonucleotides specifically silencing PIK3CD-AS2 for xenograft implantation. Our results showed that tumor originating from A549 cells grew rapidly, while LNA-PIK3CD-AS2 treatment generated smaller one (Fig. 3a). Upon harvest, we found a 2.8-fold reduction in tumor weight and a 2.1-fold decrease in tumor volume, compared with scramble control (Fig. 3b, c). qRT-PCR showed a decreased PIK3CD-AS2 mRNA level in xenograft tumors from LNA-treated side (right armpit) relative to matched control (left armpit) (Fig. 3d). Additionally, H&E, Ki-67, and TUNEL staining revealed that LNA-PIK3CD-AS2 treated tumors have a lower mitotic grade, multiple areas of cell apoptosis and smaller size of tumor nest than those from control (Fig. 3e). Hence, downregulation of PIK3CD-AS2 inhibits tumor progression of LUAD cells in vivo.

### PIK3CD-AS2 promotes LUAD progression by attenuating p53 signaling

**Fig. 4:**
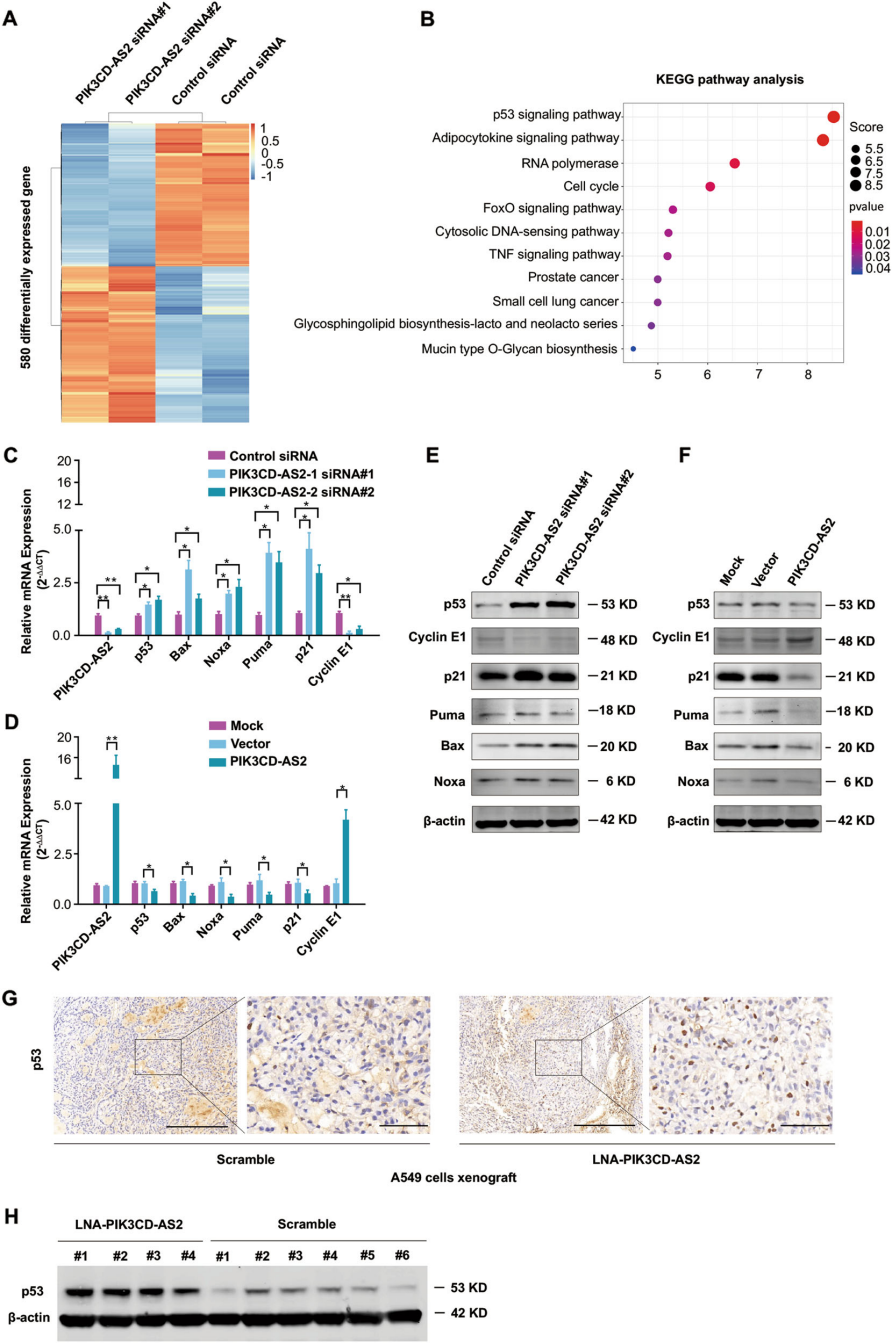
PIK3CD-AS2 represses p53 signaling activity. **a**, **b** 1 × 10^6^ A549 cells were transfected with PIK3CD-AS2 siRNA or control, respectively. Forty-eight hours after transfection, total RNA of A549 cells was harvested and analyzed by RNA sequencing (RNA-seq). Heat map depicting differentially expressed genes between si-PIK3CD-AS2 and control cells (**a**). Red: upregulated genes; green: downregulated genes. KEGG pathway enrichment analyses of RNA-seq results in the top 11 pathways according to the *P* value (**b**). The y-axis and x-axis indicate pathway name and rich score, respectively. **c**–**f** A549 cells were transfected with PIK3CD-AS2 siRNA, control siRNA, PIK3CD-AS2 plasmid or control plasmid for 48 h, respectively. p53 downstream target genes were measured by qRT-PCR (**c**, **d**) and western blot (**e**, **f**). Data are represented as means ± SD. Statistical analysis was carried out using unpaired *t*-test. *P < 0.05; **P < 0.01. **g**, **h** Representative images of p53 expression level in xenograft tumors were detected using IHC staining (**g**) and western blot (**h**). Scale bars: 500 μm and 100 μm (insets).

We next performed RNA-sequencing (RNA-seq) analysis on A549 cells upon PIK3CD-AS2 gene silencing for further mechanism investigation (GSE145018). qRT-PCR and TCGA analysis ruled out the possible effect of PIK3CD-AS2 on its host gene PIK3CD (Supplementary Fig. S5). KEGG pathways analysis indicated PIK3CD-AS2 highly affects the p53-mediated signaling (Ranking 1, *P* < 0.01, Fig. 4a, b), which is tightly associated with cell proliferation and apoptosis. Given that p53^null^ H1299 cells showed no obvious signs of promoting cell growth after PIK3CD-AS2 knockdown, we hypothesize that p53 signaling is crucial for the effect of PIK3CD-AS2 on LUAD progression.

To verify our hypothesis, we firstly determined the expression of known p53 signaling downstream genes which differentially expressed in above RNA-seq data by qPCR. As expected, after silencing PIK3CD-AS2 in A549 cells, both the mRNA and protein levels of p53, Bcl-2-associated X protein (Bax), PMA induced protein 1 (Noxa), p53-upregulated modulator of apoptosis (Puma) and p21 were upregulated, whereas the expression of cyclin E1 (CCNE) was downregulated. The opposite results were shown while PIK3CD-AS2 was overexpressed (Fig. 4c–f and Supplementary Fig. S6). Additionally, we also found an increase in p53 protein abundance in LNA-PIK3CD-AS2 administrated tumors (Fig. 4g, h).

**Fig. 5:**
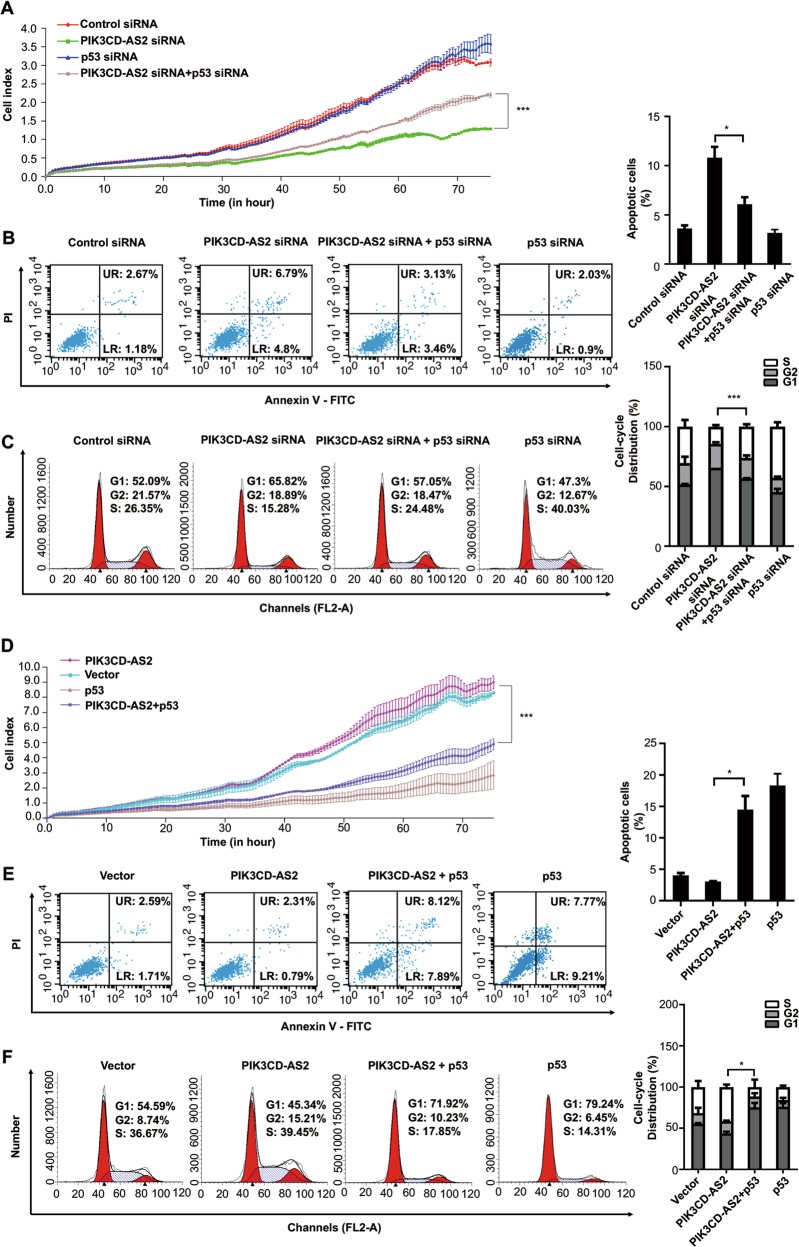
p53 rescues PIK3CD-AS2-induced LUAD cells proliferation and apoptotic resistance. **a**–**c** A549 cells were transfected with PIK3CD-AS2 siRNA, control siRNA, and p53 siRNA as the rescued group. After 24 h of transfection, cells were seeded as explained in Fig. 2c and monitored by RTCA for 90 h (**a**). ***P < 0.001 determined by unpaired *t*-test. After 48 h of transfection, cells were harvested and measured for apoptosis (**b**) and cell cycle phase distribution (**c**) using flow cytometry. **d**–**f** A549 cells were transfected with PIK3CD-AS2 expression plasmid, control plasmid, and p53 plasmid as the rescued group. Cells proliferation were monitored by RTCA (**d**). ***P < 0.001 determined by unpaired *t*-test. The apoptosis (**e**) and cell cycle phase distribution (**f**) were assessed by flow cytometry. Values represent mean ± SD of three independent experiments. Statistical analysis was carried out using unpaired *t*-test. *P < 0.05; ***P < 0.001.

Next, we confirmed transfection of p53 siRNA partially rescued cell function caused by si-PIK3CD-AS2 (Fig. 5a–c). Similarly, the administration of p53 plasmid improved PIK3CD-AS2 overexpression induced phenotypes recovery, including cell proliferation and apoptosis (Fig. 5d–f). Based on these results, we conclude that the impaired p53 signaling contributes to the biological function of PIK3CD-AS2 in regulating LUAD progression.

### PIK3CD-AS2 suppresses p53 signaling via protecting YBX1 protein from ubiquitination and degradation

**Fig. 6:**
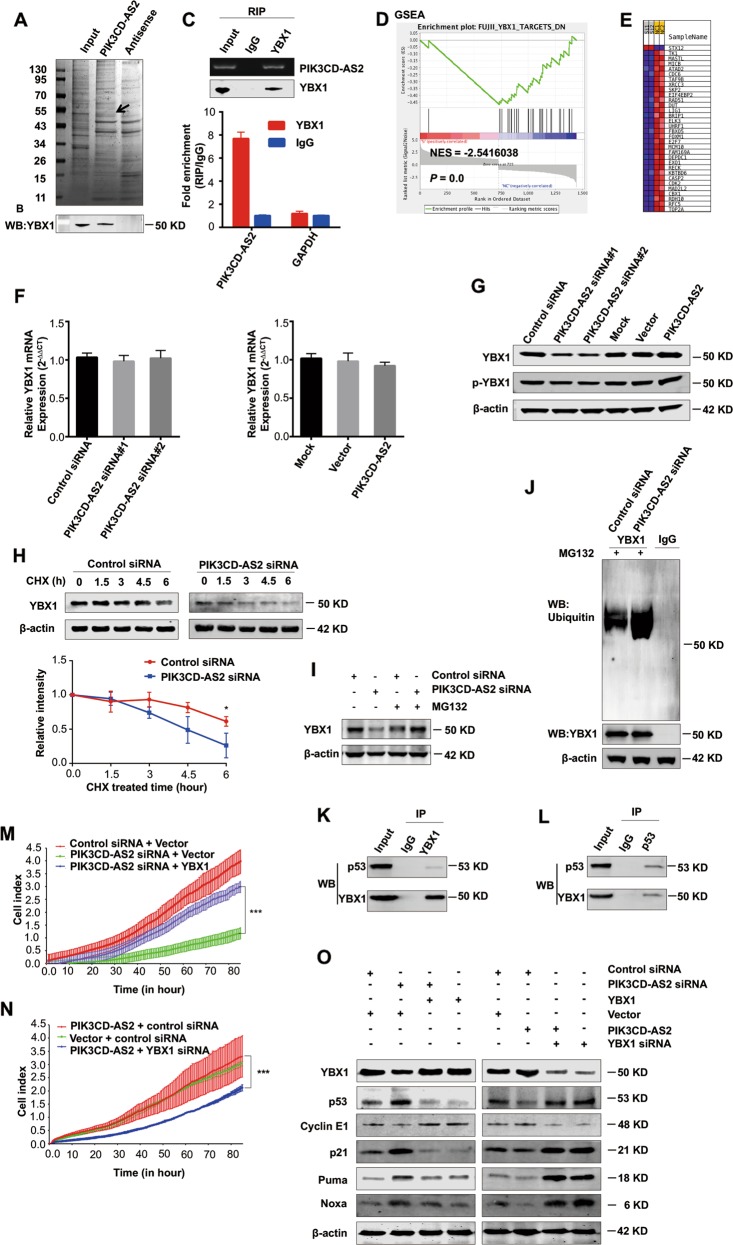
PIK3CD-AS2 suppresses p53 signaling through directly binding to and stabling YBX1 protein. **a** Total lysate from A549 cells was used as a positive control, or pulled down with biotin-labeled PIK3CD-AS2. As a negative control, the cell lysate was incubated with a PIK3CD-AS2 antisense sequence. Representative image of silver-stained PAGE gel showing separated proteins. **b** Y-box binding protein 1 (YBX1) screened by mass spectrometry (peptide coverage value is 5) was validated using western blot. **c** qRT-PCR analysis of RNA enrichment in the RIP assay using the YBX1 antibody in A549 cells. Agarose gel of qRT-PCR products for PIK3CD-AS2 (Top). The size of PCR products was ~75 base pair. YBX1 was detected by western blot (Middle). mRNA levels in immunoprecipitated samples, corrected for mRNA levels in input (Bottom). GAPDH was used as negative control for YBX1 binding. **d**, **e** GSEA enrichment plots (**d**) and heat map (**e**) of differentially expressed genes (data shown in Fig. 4a) belonging to the YBX1 targets with si-PIK3CD-AS2 treatment. The bar-code plot indicates the position of genes on the expression data rank-sorted by its association with PIK3CD-AS2, with red and blue colors indicating overexpression and underexpression in the mRNA. **f**, **g** A549 cells were treated as explained in Fig. 4c and harvested for YBX1 expression analysis by qRT-PCR (**f**) and western blot (**g**). Values represent mean ± SD of three independent experiments. **h**–**j** A549 cells were transfected with PIK3CD-AS2 siRNA or control. Forty-eight hours after transfection, some of cells were treated with cycloheximide (CHX; 10 µg/mL) for 0, 1.5, 3, 4.5 or 6 h, and then measured by western blot (**h**). Graph shows protein quantification normalized to β-actin (mean ± SD). *P < 0.05 was determined by unpaired *t*-test. Some of cells were left alone or treated with MG132 (30 µM) for 6 h, and then analyzed for YBX1 expression by western blot (**i**), and the ubiquitination levels of endogenous YBX1 using immunoprecipitation assay (**j**). **k**, **l** Total protein lysate from A549 cells was extracted for immunoprecipitation using anti-YBX1 antibody (**k**) or anti-p53 antibody (**l**). **m**–**o** A549 cells were transfected with PIK3CD-AS2 siRNA, PIK3CD-AS2 plasmid or control, respectively. Some cells were also cotransfected with YBX1 siRNA or YBX1 plasmid. After 24 h of transfection, some cells were seeded as explained in Fig. 2c and monitored by RTCA for 90 h (**m**, **n**). ***P < 0.001 determined by unpaired *t*-test. After 48 h of transfection, the expression of p53 pathway molecules was assessed by western blot (**o**).

To figure out the underlying mechanism of PIK3CD-AS2 blocking p53 activation, we performed RNA pull-down assay and detected a unique band of ~50 kDa to PIK3CD-AS2 (Fig. 6a). Using mass spectrometry, western blot and RNA immunoprecipitation (RIP) assay, we confirmed the physical interaction between PIK3CD-AS2 and Y-box binding protein 1 (YBX1), which is a p53 negative regulator (Fig. 6b, c)^17^. A gene set enrichment analysis (GSEA) using the above RNA-seq data in Fig. 4a was shown that silencing PIK3CD-AS2 resulted in YBX1 targeted gene expression change (Fig. 6d, e). These data suggested that PIK3CD-AS2 regulated YBX1.

Subsequently we found that PIK3CD-AS2 only affects the YBX1 protein level but not mRNA level. Silencing PIK3CD-AS2 decreased both YBX1 and phosphorylated YBX1 (p-YBX1) protein levels whereas ectopic overexpression elevated them (Fig. 6f, g). However, the ratio of p-YBX1/YBX1 did not change (Supplementary Fig. S7). These data indicated that PIK3CD-AS2 regulated YBX1 protein at the translational or posttranslational level, but not via its phosphorylation.

To figure out how PIK3CD-AS2 affects YBX1 protein stability, we treated PIK3CD-AS2 silenced A549 cells with the protein synthesis inhibitor cycloheximide (CHX) for 0, 1.5, 3, 4.5, and 6 h, respectively. It is evident that silencing PIK3CD-AS2 shrank the half-life of endogenous YBX1 (Fig. 6h), decreasing protein stability over the time, indicating PIK3CD-AS2 restores YBX1 protein. It was previously reported YBX1 could be in part regulated by post-translational modifications, such as E3 ubiquitin ligase mediated ubiquitination^18,19^. We then found that in the presence of MG132, a proteasome inhibitor, YBX1 protein level was almost rescued compared to si-PIK3CD-AS2 group, suggesting that silencing PIK3CD-AS2 leads to the ubiquitination degradation of YBX1 (Fig. 6i). In addition, we observed lower PIK3CD-AS2 expression led to higher ubiquitination of YBX1 in an endogenous ubiquitination assay (Fig. 6j). These results demonstrated that PIK3CD-AS2 protects YBX1 from ubiquitination and degradation.

Next, our results of co-immunoprecipitation assays (co-IP) confirmed the combination between YBX1 and p53 (Fig. 6k, l), which is consistent with previously reports^20,21^. Besides, PIK3CD-AS2 knockdown did not affect p53 protein stability using CHX or MG132 treatment, respectively (Supplementary Fig. S8). Upon YBX1 targeted rescue experiments, cells in PIK3CD-AS2 knockdown group showed a proliferation recovery after YBX1 expression plasmid transfection. Similarly, high expression of YBX1 returned activated p53 downstream genes partially to basal levels. In parallel, we also observed decreased cell growth and rescued p53 signaling in PIK3CD-AS2 plasmid and si-YBX1 co-transfected cells (Fig. 6m–o). Consequently, these results indicated that PIK3CD-AS2 inhibits p53 signaling via binding with YBX1 and protecting it from proteasomal degradation.

### PIK3CD-AS2 promotes tumor growth via decreasing p53 level in patient-derived xenograft models and results in poor prognosis

**Fig. 7:**
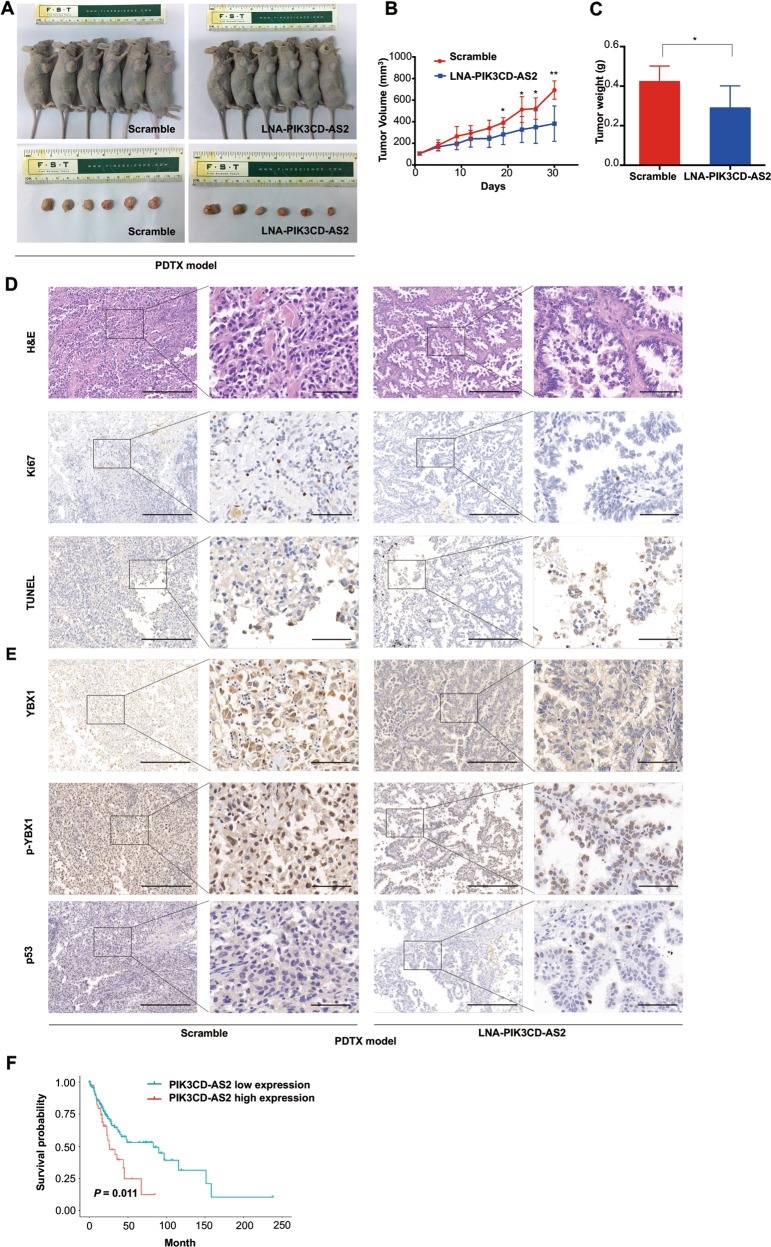
Targeting PIK3CD-AS2 ameliorates LUAD associated phenotypes in p53 wild-type PDTX mice. Tumors of PDTX models derived from p53 wild-type samples of six patients were intratumorally injected with LNA-PIK3CD-AS2 or scramble control twice weekly for four weeks. The mice were killed and analyzed four weeks after LNA administration. **a** Representative images of mice (top) and xenograft tumors (bottom) were shown. *n* = 6. **b**, **c** Tumor volume (**b**), and tumor weights (**c**) were quantitative analyzed. Data are shown as means ± SD. Statistical analysis was performed using unpaired *t*-test. *P < 0.05; **P < 0.01. **d**, **e** Xenograft tumor sections were measured for H&E staining and the expression of Ki67, TUNEL (**d**), YBX1, p-YBX1 and p53 (**e**). **f** Kaplan–Meier analysis of disease-free survival curve of p53 wild-type LUAD patients in TCGA datasets based on PIK3CD-AS2 expression (log rank test, *P* = 0.011).

To evaluate the clinical and translational relevance of PIK3CD-AS2 in LUAD and validated PIK3CD-AS2/YBX1/p53 axis in vivo, we established 12 representative LUAD patient-derived tumor xenograft (PDTX) models, of which tumors originated from six p53 wild-type surgical samples. Consistent with A549 cells in nude mice model, the reduced tumor growth was also observed in LNA knockdown groups, showing a decrease of xenograft tumor volume over a period of one month (Fig. 7a). The line chart indicated that the volume ratio of LNA-PIK3CD-AS2 treated tumor compared with control was 1.3-fold lower as early as 18 days following injection and reached 1.8-fold at the end (Fig. 7b). The tumor weight showed a 1.46-fold loss in PIK3CD-AS2 suppressed mice as well (Fig. 7c). The administration of LNA-PIK3CD-AS2 induced less disordered construction, weak expressions of Ki-67 and strong TUNEL positive areas (Fig. 7d). Moreover, the lower expression of YBX1 and p-YBX1, along with the higher level of p53 was observed in LNA-PIK3CD-AS2 tumor, suggesting PIK3CD-AS2/YBX1/p53 axis exert anti-tumor activity (Fig. 7e).

Finally, We confirmed that elevated level of PIK3CD-AS2 was remarkably correlated with poor outcome (*P* = 0.011) in p53 wild-type patients, which indirectly supported our opinion that wild-type p53 is involved in the regulatory mechanism of PIK3CD-AS2 promoting LUAD progression (Fig. 7f).

## Discussion

A number of investigations have been made in the treatment of LUAD, including novel emergence of nucleic acid drugs, which featuring the ability to be manufactured easily at moderate sized molecules and the potential to exhibit more efficacy and low toxicity. Now, more than 100 antisense oligonucleotide-based therapies have been approved in phase I clinical trials and 25% of which have reached phase II or III^22^. The continued improvement of innovative RNA-based therapeutics have offered a new entrance for a broader range of chronic diseases treatment. Many novel ncRNAs involved in cancers, especially lncRNAs are being discovered^23^. While these small yet powerful RNAs have been vastly improved, the issues of how to target these RNAs to the correct tissues still remain. Thus, well-characterized lncRNA facilitates further experiments and helps us gain more insights into cancer therapy.

In the present study, we used microarray screening, GEO and TCGA datasets to select and identify a novel lncRNA, PIK3CD-AS2, as a candidate oncogene in human LUAD. Our findings suggest that high expression of PIK3CD-AS2 is shown in tumor tissues, associated with cancer size, histological differentiation, as well as shorter survival. Functionally, we verified that loss of PIK3CD-AS2 could dramatically inhibit LUAD cell proliferation, accelerate cell-cycle arrest and promote apoptosis in vitro and substantially suppress tumor formation in vivo. Interestingly, in the cell assay, we observed that the proto-oncogenic role of PIK3CD-AS2 in p53^wt^ A549 cells failed to occur in p53^null^ H1299 cells, suggesting the differences between these two cell lines could be responsible for the influence by PIK3CD-AS2. PIK3CD-AS2 is an antisense transcription, originated from the opposite strand of protein-coding gene, PIK3CD. It is reported that antisense lncRNAs could operate as protein-coding gene modulators, leading to gene promoter activation or posttranscriptional regulation by controlling mRNA and protein stability. Using RNA-seq, bioinformatics analysis, as well as multiple-genes validation set, we speculated that p53 signal pathway might be involved in the molecular mechanism of PIK3CD-AS2. This hypothesis also explained why PIK3CD-AS2-mediated cell proliferation failed in p53^null^ H1299 cells.

The transcription factor p53 is a tumor suppressor, activating downstream targets to trigger cell cycle arrest, apoptosis^24^. It has been suggested that several lncRNAs participate in p53 regulatory network and serve as p53 regulators or effectors^25^. A recent global transcriptome study identified that sixteen p53 target lncRNAs forming a pathway web, constitute tumor suppressor signature with high diagnostic power^26^. Hung and colleagues previously demonstrated that DNA damage induces lncRNA PANDA expression in a p53-dependent manner^27^. Other studies have shown that lincRNA-p21 functions as a repressor in the p53 pathway. The binding of lincRNA-p21 to hnRNP-K provides a regression selectivity to p53 induced genes through directly targeting their promoters or lowering their translation^28–30^. Available evidences pointed that lncRNAs are also capable of posttranscriptionally regulating p53. The lincRNA-RoR suppresses p53 translation via interacting with hnRNP I, meanwhile, it creates a feedback loop with core transcription factors to regulate both the activity of p53 protein and the expression of itself^31^. Results from our further gene-silencing and ectopic expression assay and cell rescue experiment revealed that PIK3CD-AS2 gives rise to a responding inactivation of p53 downstream genes. It is possible that PIK3CD-AS2 acts as a p53 gene regulator.

Subsequently, using RNA pull-down and RIP assay, we showed a combination between PIK3CD-AS2 and YBX1, which may result in p53 pathway repression. Further experiments indicated that silence of PIK3CD-AS2 aggravated YBX1 ubiquitination levels, leading to its degradation. YBX1 is a well-known oncoprotein linked to cancer progression, including breast cancer, hepatocellular carcinoma, gastrointestinal cancer and prostate cancer^32–34^. Growing evidence reported that YBX1 can inactivate p53 protein through epigenetically regulating or directly forming a complex^35^. It is conceivable that PIK3CD-AS2/YBX1 interaction masks YBX1 ubiquitination site, protecting it from degradation and, thus, reduces basal levels of p53, as well as its downstream gene activities. Indeed, we demonstrated that YBX1 can bind to p53 protein. Our finding that cell proliferation and p53 function inhibited by PIK3CD-AS2 is rescued by YBX1 provides solid evidence to support the notion that PIK3CD-AS2-mediated suppression of p53 occurs via PIK3CD-AS2/YBX1 interaction.

Previous research has reported that p53 is mutated in around half of human tumors. Mutant p53 initiates cancer genesis and development. Nonetheless, the other half tumors carry wild-type p53, and it is reasonable to assume that p53 function is suppressed in most of them. If so, sustaining p53 expression and activity would contribute to tumor treatment. Using human p53 wild-type LUAD derived PDTX model, we showed a dramatical tumor shrink following PIK3CD-AS2 inhibition. The analysis of TCGA data suggested lower PIK3CD-AS2 expression has a better prognosis in p53 wild-type patients. These data indicated the way of targeting PIK3CD-AS2 to restore p53 might be an alternative cancer therapeutic strategy.

## Materials and methods

### Patients and tissues samples

A total of 92 paired primary LUAD tissues and adjacent non-tumor tissues were obtained from patients who received thoracic surgery at the Department of Thoracic Surgery, Jiangsu Cancer Hospital (Jiangsu, China). None of the patients received anti-cancer treatment before surgery, including chemotherapy or radiotherapy. All samples were confirmed by experienced pathologists and obtained from biobank of Jiangsu Cancer Hospital (Jiangsu Institute of Cancer Research, Nanjing Medical University affiliated Cancer Hospital). All patients had signed informed consent for donating their samples.

### Cell culture and transfection

All cell lines (A549, H1299, H1975, PC9, H358, SPC-A1, and HBE1) were obtained from the Cell Bank of the Chinese Academy of Sciences (Shanghai, China). A549, H1299, H1975, PC9, H358 cells were cultured in RPMI 1640 medium (KeyGene, Nanjing, China), SPC-A1 and HBE1 cells were cultured in DMEM medium (KeyGene, Nanjing, China), with 100 units/mL penicillin and 100 units/mL streptomycin and 10% FBS (Thermo Fisher Scientific, MA, USA) at 37 °C in an incubator with 5% CO_2_. Authentication of cells were verified by STR profiling and mycoplasma contamination of cells were tested using MycoBlue^TM^ mycoplasma detector (Vazyme, Nanjing, China). Transfections were performed using the Lipofectin reagent (Thermo Fisher Scientific) when cells were seeded at 50–80% confluence, according to the manufacturer’s protocol. For siRNA infection, cells were prepared to 30–50% confluence. Forty-eight hours after transfection, the cells were rinsed twice with phosphate-buffered saline and harvested for next experiments. CHX and MG132 were purchased from MedChem Express (NJ, USA).

### siRNAs and plasmid

Predesigned siRNAs for human PIK3CD-AS2 and scramble negative control siRNA were obtained from Thermo Fisher Scientific. YBX1 siRNA (sc-38634), p53 siRNA (sc-29435) and control siRNA were purchased from Santa Cruz Biotechnology (Santa Cruz, CA, USA). The recombinant PIK3CD-AS2 expression plasmid containing full-length human PIK3CD-AS2 cDNA was constructed by GENEray Biotechnology Shanghai, China). Plasmid pcDNA3 p53 wild-type was a gift from Dr. Yu Cao. The pcDNA-YBX1 overexpression plasmid was prepared by ligating the YBX1 sequence into the BamHI/EcoRI sites of pcDNA3.1(+) vector (Thermo Fisher Scientific) as previously described^36^.

### Tissue microarray (TMA) and CISH

The TMA construction method and CISH was performed as our previously reported^16^. The construction of TMA contained pairs of lung adenocarcinoma tumor tissues and adjacent non-tumor tissues. The expression of PIK3CD-AS2 was detected through RNA CISH using PIK3CD-AS2 specific probe (5′-ACCTGTGCTCTCATCTCTTGCT-3′).

### Fluorescence in situ hybridization (FISH)

The cellular localization of PIK3CD-AS2 was detected by FISH as our previously reported^14^. Briefly, cells were fixed in 4% formaldehyde in PBS (pH 7.4). After hybridized with PIK3CD-AS2 specific probe, double-digoxigenin-labeled (DIG-labeled) LNA detection probes (80 nM, Exiqon, Vedbaek), at 55 °C for 2 h, the cells were incubated at RT for 1 h with an anti-DIG-fluorescein monoclonal antibody (catalog 11207741910, Roche Life Science). Subsequently, cells were counterstained with DAPI and imaged using an AxioVision Zeiss fluorescence microscope.

### RNA pull-down

The pcDNA-PIK3CD-AS2 vector and negative control PIK3CD-AS2 antisense plasmid was linearly cut by restriction enzymes. PIK3CD-AS2 and the antisense control was next transcribed and purified in vitro using mMESSAGE mMACHINE kit (Thermo Fisher Scientific) and RNeasy Mini kit (Qiagen, CA, USA), according to the manufacturer’s protocol. After that, we biotinlabeled the 3′ end of PIK3CD-AS2 referencing to the instruction of Pierce RNA 3′ End Desthiobiotinylation Kit (Thermo Fisher Scientific). The next pull-down assay was performed as manual instruction using a Pierce Magnetic RNA-Protein Pull-Down Kit (Thermo Fisher Scientific). Briefly, desthiobiotin-labeled RNA is bound to the Streptavidin Magnetic Beads, and then mixed with whole cell lysates. The RNA-protein complex was isolated from magnetic beads by Biotin Elution Buffer and boiled for mass spectrometry analysis and western blot.

### RNA immunoprecipitation (RIP)

RIP experiments were carried out using a EZ-Magna RIP kit (Millipore, MA, USA) according to the manufacturer’s instructions. Briefly, the cells were extracted and lysed with RIP lysis buffer. Magnetic beads were washed and resuspended in 100 μL RIP wash buffer, followed by incubation with YBX1 antibody for 30 min at room temperature. The beads-antibody complex was then mixed with 900 μL RIP immunoprecipitation buffer and 100 μL cell lysis at 4 °C overnight. Next day, proteinase K was added to each immunoprecipitated products, incubated at 55 °C for 30 min to digest the protein. After centrifuging, 400 μL phenol: chloroform: isoamyl alcohol was used to extract total RNA subjected to further qRT-PCR analysis.

### Subcellular fractionation

The subcellular localization of PIK3CD-AS2 was investigated using PARIS^TM^ Kit according to the manufacturer’s protocol (Thermo Fisher Scientific).

### Co-immunoprecipitation (Co-IP)

The Co-IP assay was performed by Pierce Co-immunoprecipitation kit according to the manufacturer’s instructions (Thermo Fisher Scientific). In brief, YBX1 (ab76149, Abcam, MS, USA) and p53 (sc-126, Santa Cruz) antibody was incubated with AminoLink Plus Coupling Resin for 90 min at room temperature, respectively. Meanwhile, Cell lysate was prepared in IP buffer and centrifuged for 10 min at 13,000 × *g* at 4 °C. The supernatant was added to the Pierce Spin Column to incubate overnight at 4 °C. Next day, total precipitated protein was eluted and subjected to western blot analysis.

### Western blot

Western blot was performed according to the routine protocol. Cells were washed twice in chilled PBS and lysed. Supernatants derived from cell extracts were separated on 10% SDS-PAGE gel, followed by transferred to a PVDF membrane. After blocking in 5% nonfat dry milk, the PVDF membrane was incubated with diluted primary antibodies. The information of primary antibodies is listed in Supplementary Table S2. IRDye 800CW goat anti-mouse or IRDye 680CW goat anti-rabbit (Li-Cor Biosciences, NE, USA) secondary antibody was used at 1:10,000 dilution. The signal was detected using an Odyssey scanner (Li-Cor Biosciences).

### qRT-PCR analysis

Total RNA was extracted from tissues and cells using TRIzol reagent (Thermo Fisher Scientific) and qRT-PCR was performed to target RNA using Fast SYBR® Green Master Mix (Thermo Fisher Scientific) on a QuantStudio 6 (Applied Biosystems, CA, USA) as directed by the manufacturer. Forward and reverse primer sequences for specific genes listed in Supplemental Table S3.

### Cell proliferation assay

Cell proliferation was detected by real time xCELLigence analysis system (RTCA) according to the manufacturer (ACEA Biosciences, CA, USA). After transfected with siRNA, expression plasmid or scramble control, respectively, 5 × 10^3^ cells were seeded to each well of E-Plate and incubated at 37 °C with 5% CO_2_ with proliferation monitoring every 15 min for at least 90 h.

### Flow cytometry

For cell cycle distribution analysis, 1 × 10^5^ cells were fixed in ice-cold 70% ethanol before staining with propidium iodide (PI). The proportion of each phase of the cell cycle was calculated by FACS analysis equipped with Cell Quest software (BD Biosciences, CA, USA). Cell apoptosis was analyzed by an FITC annexin V detection kit with PI according to the manufacturer’s protocol. Briefly, cells were suspended in 1× binding buffer at a concentration of 1 × 10^6^ cells/mL. The cell suspension (100 μL) was then transferred to a flow meter tube, mixed with 5 μL FITC annexin V and 10 μL PI, and incubated for 20 min at RT in darkness. Samples were analyzed by flow cytometry within 1 h.

### EdU proliferation assay

Cells were cultured in 96-well plates in complete media until 80–90% confluent and then treated with 50 μM 5-ethynyl-2′-deoxyuridine (EdU) for 6 h to measure proliferation according to the manufacturer’s instructions using an EdU DNA Cell Proliferation Kit (RiboBio, Guangzhou, China).

### Cell migration and invasion assays

For invasion assay, 4 × 10^4^ cells were seeded on the upper Matrigel-coated chambers (8-μm pore) with serum-free RPMI 1640 medium, the lower wells were filled with 600 μL medium containing 10% FBS. After the chambers were incubated at 37 °C in an incubator with 5% CO_2_ for 24 h, the invasiveness was calculated by the number of cells invaded through the chamber and adhered to the bottom of the membrane. For wound healing assays, cells were transfected with PIK3CD-AS2 siRNA or control, and the wounds were made using a pipette tip. The cells were cultured in low-serum medium (0.1% FBS), and pictured after 36 h of wounds.

### Animal models

The animal care and the study procedures were approved by Animal Experimentation Ethics Committee of Nanjing Medical University (2015–174). Five-week old NOD/SCID mice were purchased from Vital River Laboratories (Beijing, China), maintained in facilities at the center of animal experiments of Nanjing Medical University. 5 × 10^6^ cells were engrafted into the left and right armpits of six male BALB/C nude mice, which subsequently receiving two injections of LNA-PIK3CD-AS2 or negative control the first week, spaced two days apart, and weekly injections of the treatment thereafter. Mice were sacrificed after six weeks, and tumor size and weight were measured. Frozen or paraffin-embedded tumor samples were analyzed using qRT-PCR or immunohistochemistry, respectively. PDTX model was performed as previously described^16^. Primary LUAD tumor was cut into 1 mm^3^ fragments in 0.1 mL 50% Matrigel Basement Membrane Matrix (BD Biosciences) and directly implanted into the subcutaneous space. After second generation, PDTX tumors were received genomic and histological examination. Mice with approximately 200 mm^3^ tumor were randomized into experimental group, giving LNA-PIK3CD-AS2 injection (1.5 mg/kg) twice a week, or scramble control with same dosage and treatment frequency. Tumor size was measured every week using calipers. The mice were euthanized after one month, and the tumors were excised and snap-frozen or paraffin embedded for further analysis.

### Immunochemistry

Five micrometer sections from formalin-fixed paraffin-embedded tumor tissues were deparaffinized, rehydrated, and rinsed in distilled water. Following antigen retrieval in boiling 0.1 M citrate (PH 6.0) buffer for 10 min, the slides were then probed with antibodies against p53, YBX1, p-YBX1 and Ki-67. The detail of antibodies is listed in Supplementary Table S2. The tissue sections were also received H&E and TUNEL staining (Promega, WI, USA). The images were observed on a Zeiss Axio fluorescent microscope and evaluated by pathologists blind to the study design.

### Statistical analysis

Statistical analysis was carried out using GraphPad Prism and SPSS 20.0 (SPSS, Inc., IL, USA). Normal distribution of the data was tested. Data were presented as means ± SD. Differences between groups were assessed by two-tailed Student’s *t*-test and paired *t*-test. The association between clinical characteristics and PIK3CD-AS2 expression was analyzed by the chi-square test. Survival curves were plotted using Kaplan–Meier method, and differences between survival curves were tested using the log-rank test. Statistical significance was considered with a *P* value less than 0.05. The sample size for patients and animal model were determined as previously described^14–16^.

## Supplementary information

Supplementary figure legends

Supplementary table 1

Supplementary table 2

Supplementary table 3

Supplementary figure 1

Supplementary figure 2

Supplementary figure 3

Supplementary figure 4

Supplementary figure 5

Supplementary figure 6

Supplementary figure 7

Supplementary figure 8
