# Supplementary material for: The long non-coding RNA PIK3CD-AS2 promotes lung adenocarcinoma progression via YBX1-mediated suppression of p53 pathway
Source: Oncogenesis. 2020 Mar 12;9(3):34. doi: 10.1038/s41389-020-0217-0 (PMC7067885; doi:10.1038/s41389-020-0217-0)
Supplement: Supplementary file 1 — Supplementary figure legends [file 41389_2020_217_MOESM1_ESM.docx]

**Supplementary Materials**

**The long non-coding RNA PIK3CD-AS2 promotes lung adenocarcinoma progression via YBX1-mediated suppression of p53 pathway**

Xiufen Zheng^1,2#^, Junying Zhang^3#^, Tian Fang^4#^, Xiaoxiao Wang^5#^, Siwei Wang^1^ Zhifei Ma^1^, Youtao Xu^1^, Chencheng Han^1^, Mengting Sun^6^, Lin Xu^1^, Jie Wang^1,6,7*^, Rong Yin^1,6,7*^

^1^Department of Thoracic Surgery, Jiangsu Key Laboratory of Molecular and Translational Cancer Research, Jiangsu Cancer Hospital, Jiangsu Institute of Cancer Research, The Affiliated Cancer Hospital of Nanjing Medical University, Nanjing 210009, China

^2^Department of Pharmacy, The First Affiliated Hospital of Hainan Medical University, Hainan 570102, China

^3^Clinical Cancer Research Center, Jiangsu Cancer Hospital, Jiangsu Institute of Cancer Research, The Affiliated Cancer Hospital of Nanjing Medical University, Nanjing 210009, China

^4^Department of Comparative Medicine, Jinling Hospital, Clinical School of Medical College of Nanjing University, Nanjing 210093, China

^5^GCP Research Center, Affiliated Hospital of Nanjing University of Chinese Medicine, Jiangsu Province Hospital of TCM, Nanjing 210029, China

^6^Department of Tumor Biobank, Jiangsu Cancer Hospital, Jiangsu Institute of Cancer Research, The Affiliated Cancer Hospital of Nanjing Medical University, Nanjing 210009, China

^7^Department of Science & Technology, Jiangsu Cancer Hospital, Jiangsu Institute of Cancer Research, The Affiliated Cancer Hospital of Nanjing Medical University, Nanjing 210009, China

**Supplementary Figure legends**

**Supplementary Fig. S1 PIK3CD-AS2 is overexpressed in human cancers.**

**a-c** Higher PIK3CD-AS2 expression in lung squamous (**a**), liver cancer (**b**) and kidney renal papillary cell carcinoma (KIRC; **c**) tissues than in corresponding normal tissues in TCGA datasets. Data are presented as median ± 1.5 IQR. *P* values were determined by *t*-test.

**Supplementary Fig. S2 Expression analysis of PIK3CD-AS2 transcripts in LUAD tissues.**

**a** PIK3CD-AS2 has three transcripts, in which ENSG00000231789 is the longest one with three exons. **b** Correlation between PIK3CD-AS2 exon 1 product and exon 2 product levels in LUAD tissues was analyzed (*r* = 0.96, *P* < 0.0001, Spearman’s correlation test, n = 24). **c** The expression levels of all PIK3CD-AS2 transcripts (primer was located in exon 1) and ENSG00000231789 transcript (primer was located in exon 2) in LUAD tissues were measured by qRT-PCR. **d** Two percent agarose gel of PIK3CD-AS2 transcripts cDNA fragments was generated by standard PCR. Lanes show two LUAD samples and matched normal tissues. Exon 1 represents all PIK3CD-AS2 transcripts products (177 bp). Exon 1-2 represents ENSG00000231789 specific transcript product (471bp).

**Supplementary Fig. S3 PIK3CD-AS2 has no protein coding potentiality.**

The protein coding possibility of PIK3CD-AS2 transcript was identified through the Coding Potential Assessment Tool (http://lilab.research.bcm.edu/cpat; **a**)**,** Open Reading Frame Finder (https://www.ncbi.nlm.nih.gov/orffinder/; **b**) and Coding Potential Calculator (http://cpc2.cbi.pku.edu.cn; **c**).

**Supplementary Fig. S4 PIK3CD-AS2 silencing decreases cell proliferation and invasion *in vitro*.**

**a** PIK3CD-AS2 expression level in normal human bronchial epithelial cell line (HBE1) and lung cancer cell lines (H1975, PC9, SPC-A1, H358, A549 and H1299) was determined using qRT-PCR. Data are represented as mean ± SD. *P* value was determined by unpaired *t* test. ****P* < 0.001. **b-d** A549 and H1299 cells were transfected with PIK3CD-AS2 siRNA or control siRNA, respectively. mRNA was extracted from some cells and examined for the expression of PIK3CD-AS2 using qRT-PCR after 48 hours (**b**). The proportion of invaded cells was calculated using transwell invasion assay (**c**). Values represent mean ± SD of three independent experiments. Statistical analysis was performed using unpaired *t* test. **P* < 0.05. Scale bars: 100 μm. Cell migration ability was assessed by wound healing assay (**d**). Scale bars: 50 μm.

**Supplementary Fig. S5 PIK3CD-AS2 does not influence the expression of PIK3CD.**

**a** The correlation between PIK3CD-AS2 and PIK3CD levels in LUAD tissues was analyzed (*r* = -0.15, Spearman’s correlation test, n = 24). **b** A549 and H1299 cells were transfected with PIK3CD-AS2 siRNA or control siRNA, respectively. After 48 hours, mRNA was extracted and examined for the expression of PIK3CD-AS2 and PIK3CD by qRT-PCR.

**Supplementary Fig. S6 PIK3CD-AS2 regulate the expression of p53 protein.**

A549 cells were transfected with PIK3CD-AS2 siRNA, control siRNA, PIK3CD-AS2 plasmid or control plasmid, respectively. After 48 hours, the expression of p53 protein was detected by western blot and quantified. Values represent mean ± SD of three independent experiments. *P* value was determined by unpaired *t* test. **P* < 0.05, ***P* < 0.01.

**Supplementary Fig. S7 PIK3CD-AS2 fails to impact YBX1 phosphorylation.**

A549 cells were transfected with PIK3CD-AS2 siRNA, control siRNA, PIK3CD-AS2 plasmid or control plasmid, respectively. After 48 hours, the level of YBX1 and p-YBX1 in the cell lysates was analyzed by western blot. Densitometric quantification of p-YBX1 levels relative to total YBX1 levels were shown. Values represent mean ± SD of three independent experiments.

**Supplementary Fig. S8 PIK3CD-AS2 does not affect p53 stability.**

A549 cells were transfected with PIK3CD-AS2 siRNA or control. 48 hours after transfection, some cells were treated with CHX (10 µg/mL) for 0, 1.5, 3, 4.5 or 6 hours, others were left alone or treated with MG132 (30 µM) for 6 hours. All the cells were harvested and then analyzed for p53 expression by western blot.
