# Supplementary material for: The long non-coding RNA PIK3CD-AS2 promotes lung adenocarcinoma progression via YBX1-mediated suppression of p53 pathway
Source: Oncogenesis. 2020 Mar 12;9(3):34. doi: 10.1038/s41389-020-0217-0 (PMC7067885; doi:10.1038/s41389-020-0217-0)
Supplement: Supplementary file 2 — Supplementary table 1 [file 41389_2020_217_MOESM2_ESM.docx]

**Supplementary Table S1. Association between PIK3CD-AS2 expression and clinicopathological characteristics of 92 lung adenocarcinoma patients**

| Patient characteristics | Number | PIK3CD-AS2 expression | | | | *P*-value* |
| --- | --- | --- | --- | --- | --- | --- |
|  |  | Low (%) | | High (%) | |  |
| Gender |  |  |  |  |  |  |
| Male | 67 | 32 | 69.6% | 35 | 76.1% | 0.482 |
| Female | 25 | 14 | 30.4% | 11 | 23.9% |  |
| Age (years) |  |  |  |  |  |  |
| ≥ 60 | 41 | 22 | 47.8% | 19 | 41.3% | 0.529 |
| < 60 | 51 | 24 | 52.2% | 27 | 58.7% |  |
| History of smoking |  |  |  |  |  |  |
| No | 47 | 23 | 48.9% | 24 | 52.2% | 0.835 |
| Yes | 45 | 23 | 51.1% | 22 | 47.8% |  |
| Tumor size (cm) |  |  |  |  |  |  |
| < 3 | 34 | 22 | 47.8% | 12 | 26.1% | 0.031 |
| ≥ 3 | 58 | 24 | 52.2% | 34 | 73.9% |  |
| Histological differentiation |  |  |  |  |  |  |
| Well | 25 | 18 | 39.1% | 7 | 15.2% | 0.035 |
| Moderate | 20 | 8 | 17.4% | 12 | 26.1% |  |
| Poor | 47 | 20 | 43.5% | 27 | 58.7% |  |
| Lymph node metastasis |  |  |  |  |  |  |
| No | 50 | 28 | 60.9% | 22 | 47.8% | 0.209 |
| Yes | 42 | 18 | 39.1% | 24 | 52.2% |  |
| TNM stage |  |  |  |  |  |  |
| I | 34 | 22 | 47.8% | 12 | 26.1% | 0.075 |
| II | 25 | 9 | 19.6% | 16 | 34.8% |  |
| III-IV | 33 | 15 | 32.6% | 18 | 39.1% |  |

**P* values for categorical variables are from chi-square test.
