# Supplementary material for: The long non-coding RNA PIK3CD-AS2 promotes lung adenocarcinoma progression via YBX1-mediated suppression of p53 pathway
Source: Oncogenesis. 2020 Mar 12;9(3):34. doi: 10.1038/s41389-020-0217-0 (PMC7067885; doi:10.1038/s41389-020-0217-0)
Supplement: Supplementary file 3 — Supplementary table 2 [file 41389_2020_217_MOESM3_ESM.docx]

**Supplementary Table S2. Information on antibodies used in this study**

| **Antibody** | **WB** | **IHC** | **Specificity** | **Company (catalog number)** |
| --- | --- | --- | --- | --- |
| Puma | 1:1000 | -- | Rabbit monoclonal | Abcam (ab33906) |
| Noxa | 1:1000 | -- | Rabbit monoclonal | Abcam (ab140129) |
| YBX1 | 1:1000 | 1:50 | Rabbit monoclonal | Abcam (ab76149) |
| p-YBX1 | -- | 1:1000 | Rabbit polyclonal | Abcam (ab74162) |
| p53 | 1:1000 | 1:50 | Mouse monoclonal | Santa Cruz Biotechnology (sc-126) |
| Bax | 1:1000 | -- | Rabbit monoclonal | CST (5023) |
| Cyclin E1 | 1:1000 | -- | Rabbit monoclonal | CST (4129) |
| p21 | 1:1000 | -- | Rabbit monoclonal | CST (2947) |
| Ki67 | -- | 1:200 | Rabbit monoclonal | Servicebio (GB13030-2) |
| Ubiquitin | 1:1000 | -- | Rabbit polyclonal | CST (3933S) |

Abbreviations: WB, Western blot; IHC, immunohistochemistry.
