# Supplementary material for: The long non-coding RNA PIK3CD-AS2 promotes lung adenocarcinoma progression via YBX1-mediated suppression of p53 pathway
Source: Oncogenesis. 2020 Mar 12;9(3):34. doi: 10.1038/s41389-020-0217-0 (PMC7067885; doi:10.1038/s41389-020-0217-0)
Supplement: Supplementary file 4 — Supplementary table 3 [file 41389_2020_217_MOESM4_ESM.docx]

**Supplementary Table S3.** **The sequences of the gene-specific primers used in qRT-PCR and siRNAs**

| **Gene name** | **Forward primer (5'-3')** | **Reverse primer (5'-3')** |
| --- | --- | --- |
| Primers for qRT-PCR | |  |
| PIK3CD-AS2 | GGGATCATAAATGCTTGCTGTT | CGTATTAGTTACTGGTTGCTGTT |
| p53 | CAGCACATGACGGAGGTTGT | TCATCCAAATACTCCACACGC |
| Bax | CCCGAGAGGTCTTTTTCCGAG | CCAGCCCATGATGGTTCTGAT |
| Puma | GCCAGATTTGTGAGACAAGAGG | CAGGCACCTAATTGGGCTC |
| Noxa | CGAAGATTACCGCTGGCCTA | TGAACTGTTTCTCCCCAGCC |
| Cyclin E1 | ACTCAACGTGCAAGCCTCG | GCTCAAGAAAGTGCTGATCCC |
| p21 | TGTCCGTCAGAACCCATGC | AAAGTCGAAGTTCCATCGCTC |
| β-actin | GTCATTCCAAATATGAGATGCGT | GCTATCACCTCCCCTGTGTG |
| GAPDH | GGTGAAGGTCGGAGTCAACG | TGGGTGGAATCATATTGGAACA |
| siRNA (target sequence) | |  |
| PIK3CD-AS2#1 | GCUUGCUGUUCAGUCUGCCAAGUGU | ACACUUGGCAGACUGAACAGCAAGC |
| PIK3CD-AS2#2 | UCCAUGCUUAGAGGCUCCAUCAAUA | UAUUGAUGGAGCCUCUAAGCAUGGA |
| PIK3CD-AS2#3 | UCCGGUGGCUGAUUUCUCAUCUUGG | CCAAGAUGAGAAAUCAGCCACCGGA |
| YBX1 | GGUCAUCGCAACGAAGGUU | AACCUUCGUUGCGAUGACC |
| p53 | GAAAUUUGCGUGUGGAGUATT | UACUCCACACGCAAAUUUCCT |
