# Supplementary figures and images for: The long non-coding RNA PIK3CD-AS2 promotes lung adenocarcinoma progression via YBX1-mediated suppression of p53 pathway
Source: Oncogenesis. 2020 Mar 12;9(3):34. doi: 10.1038/s41389-020-0217-0 (PMC7067885; doi:10.1038/s41389-020-0217-0)

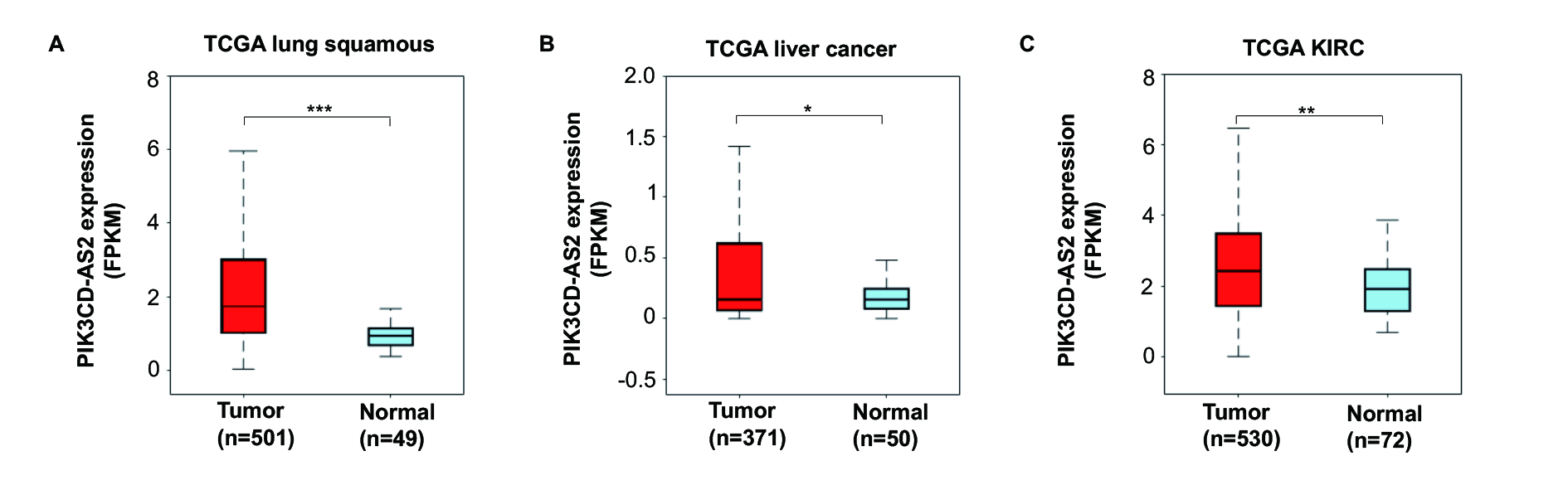

Supplement: Supplementary file 5 — Supplementary figure 1 [file 41389_2020_217_MOESM5_ESM.tif]

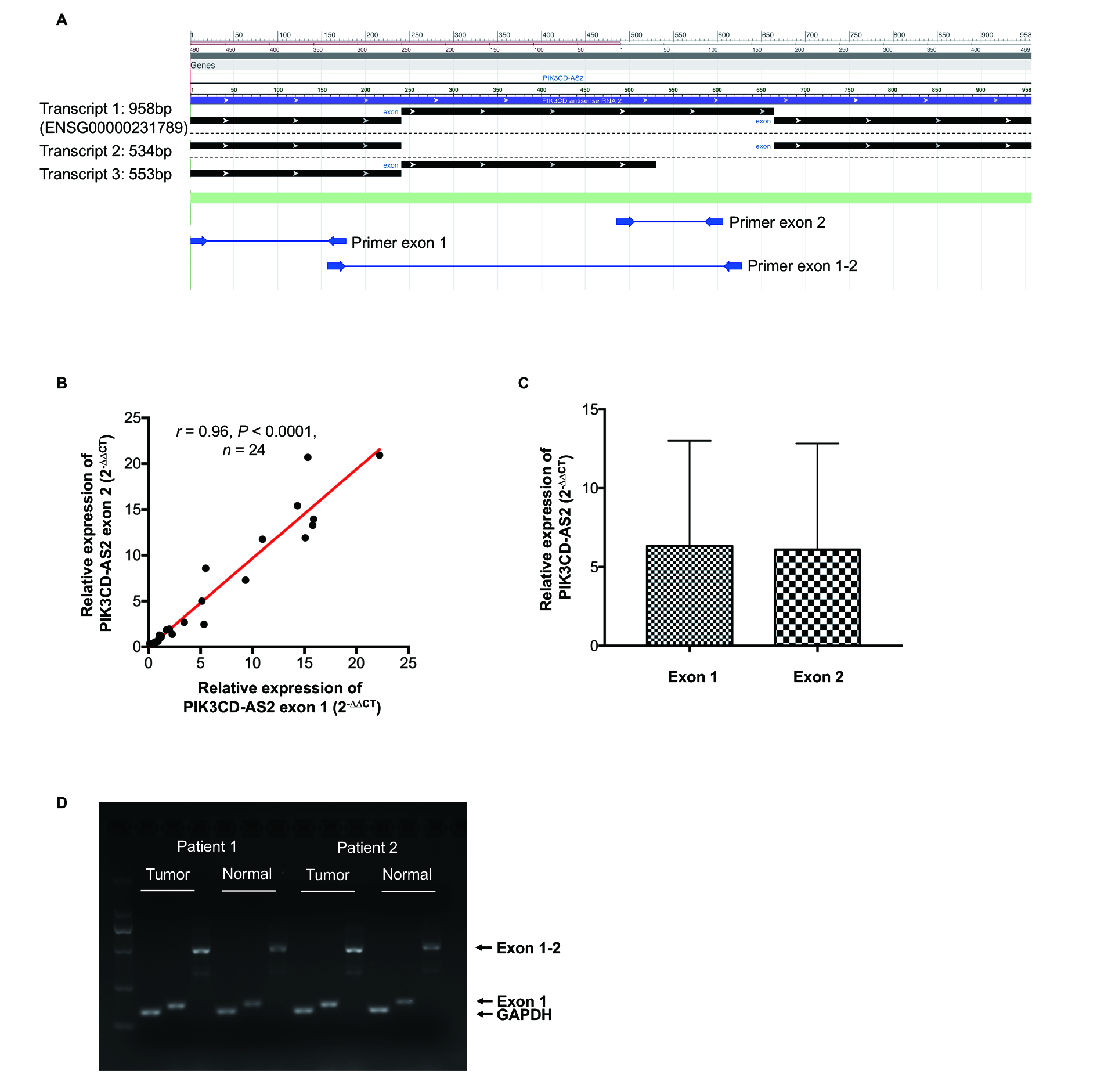

Supplement: Supplementary file 6 — Supplementary figure 2 [file 41389_2020_217_MOESM6_ESM.tif]

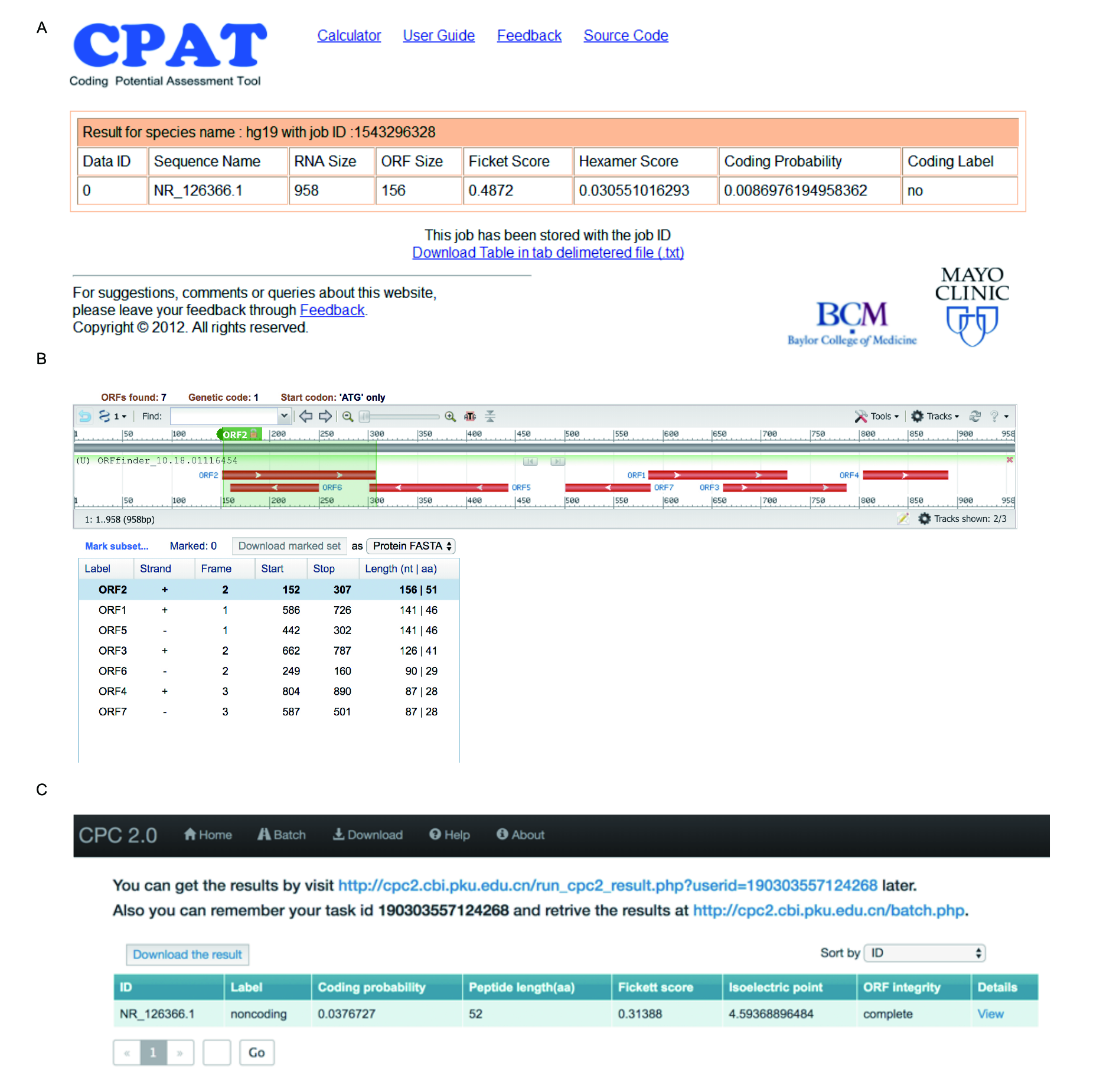

Supplement: Supplementary file 7 — Supplementary figure 3 [file 41389_2020_217_MOESM7_ESM.tif]

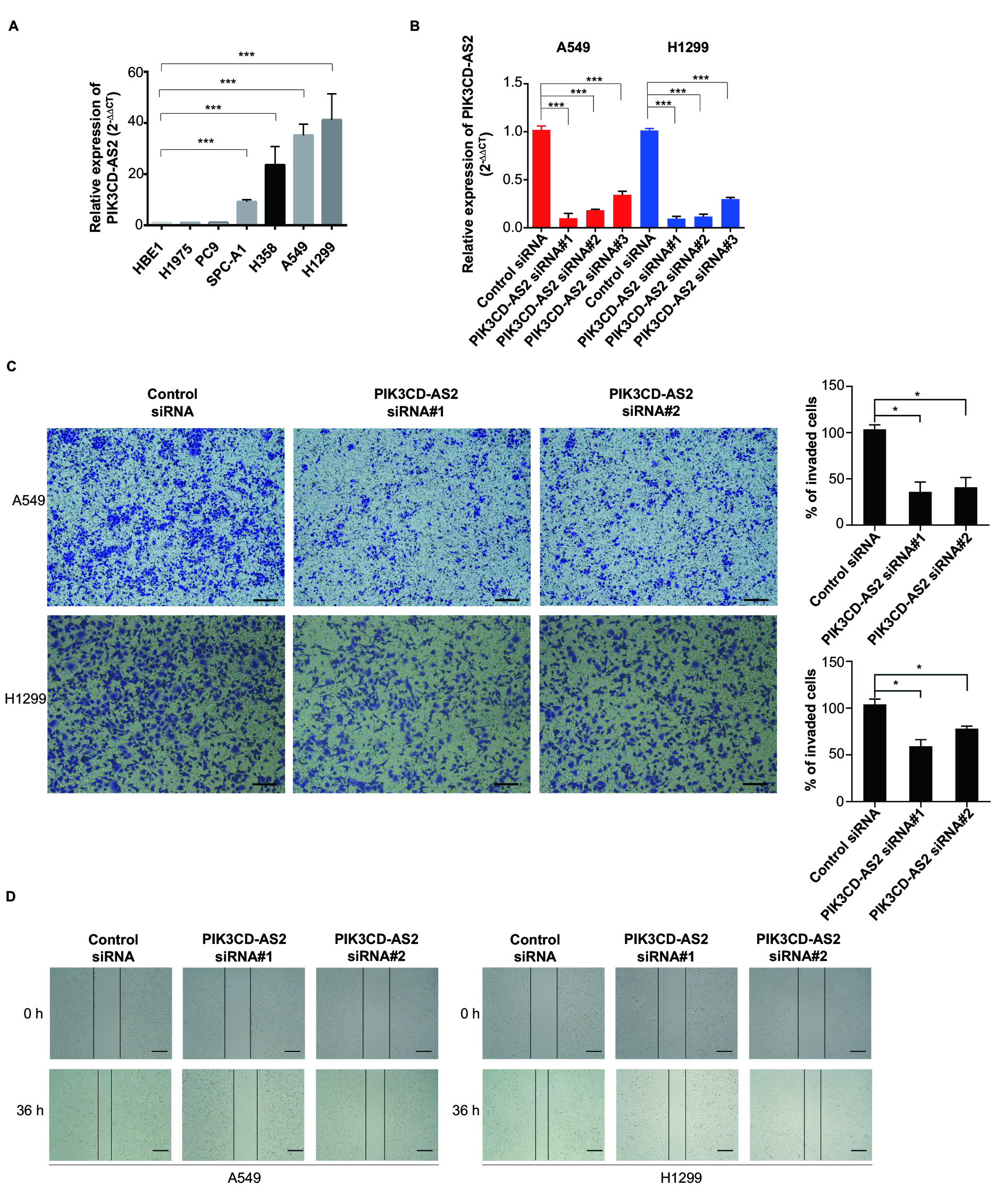

Supplement: Supplementary file 8 — Supplementary figure 4 [file 41389_2020_217_MOESM8_ESM.tif]

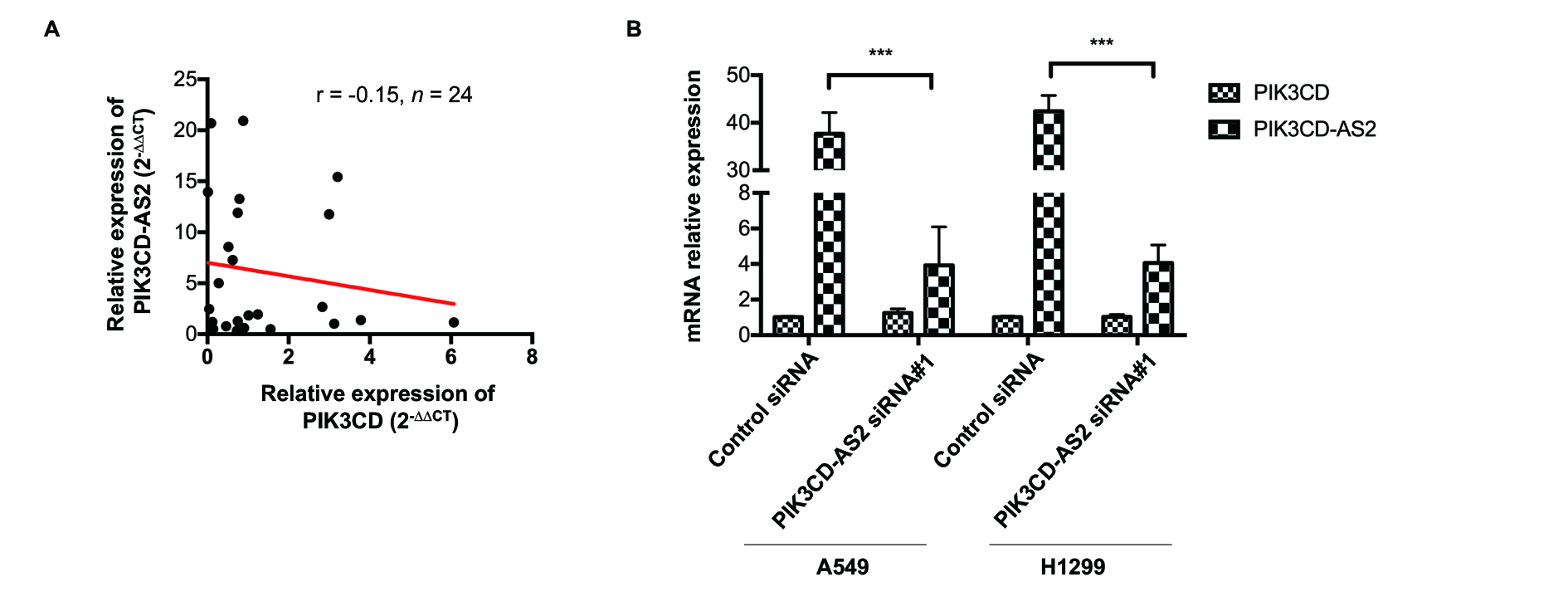

Supplement: Supplementary file 9 — Supplementary figure 5 [file 41389_2020_217_MOESM9_ESM.tif]

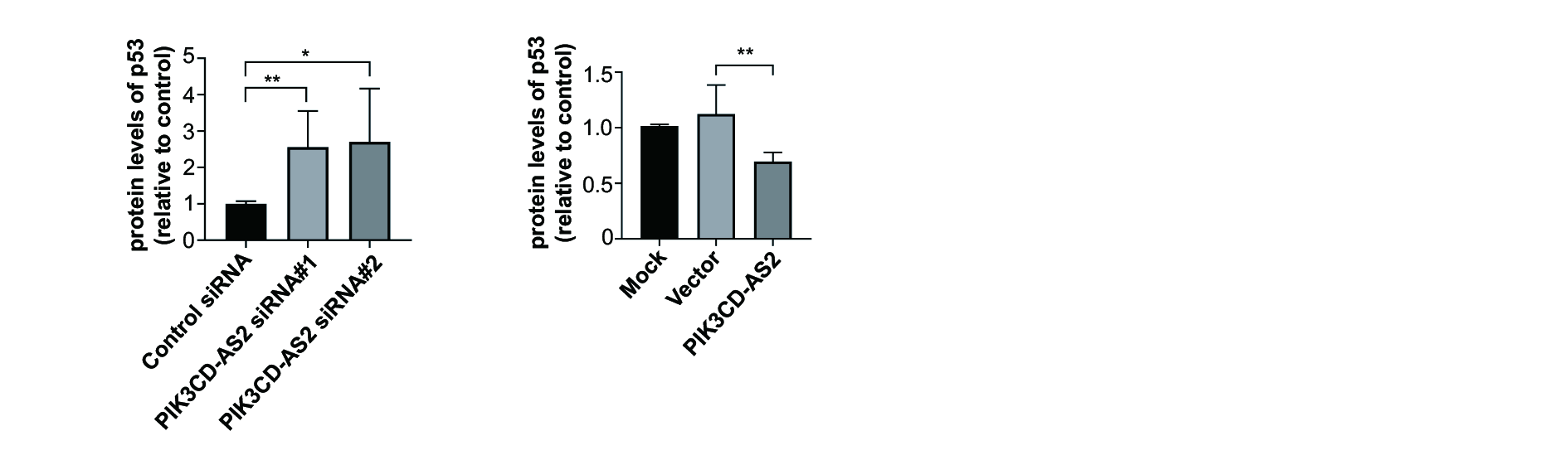

Supplement: Supplementary file 10 — Supplementary figure 6 [file 41389_2020_217_MOESM10_ESM.tif]

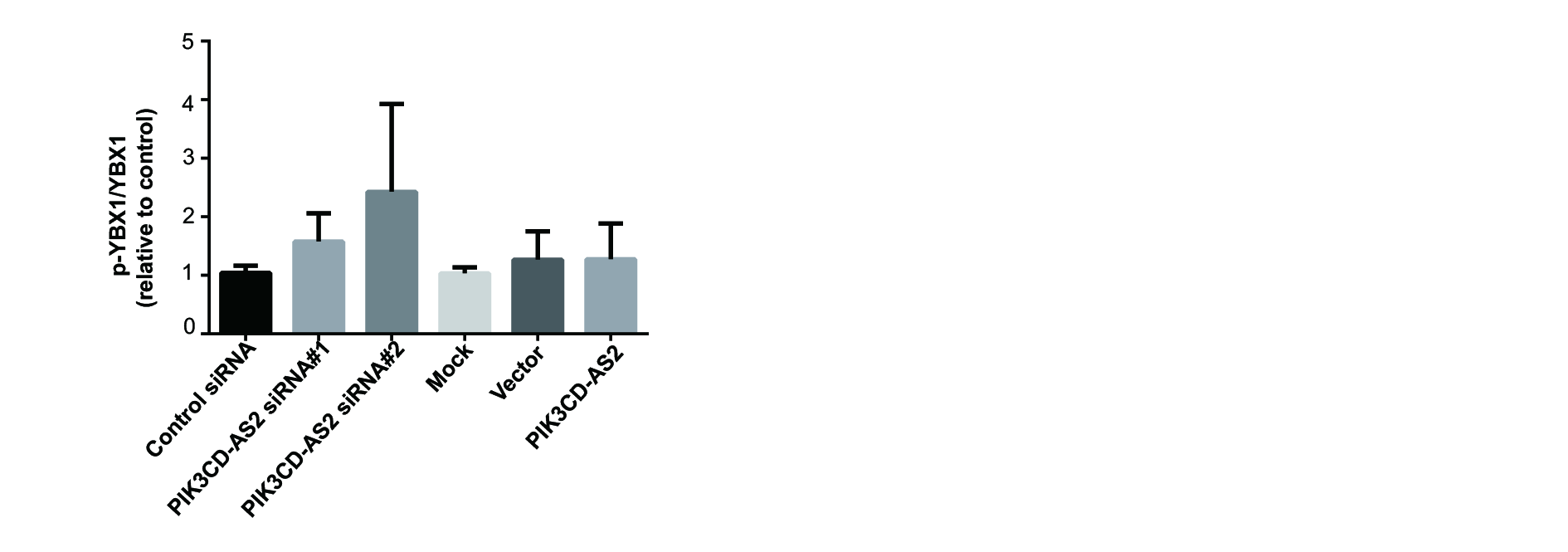

Supplement: Supplementary file 11 — Supplementary figure 7 [file 41389_2020_217_MOESM11_ESM.tif]

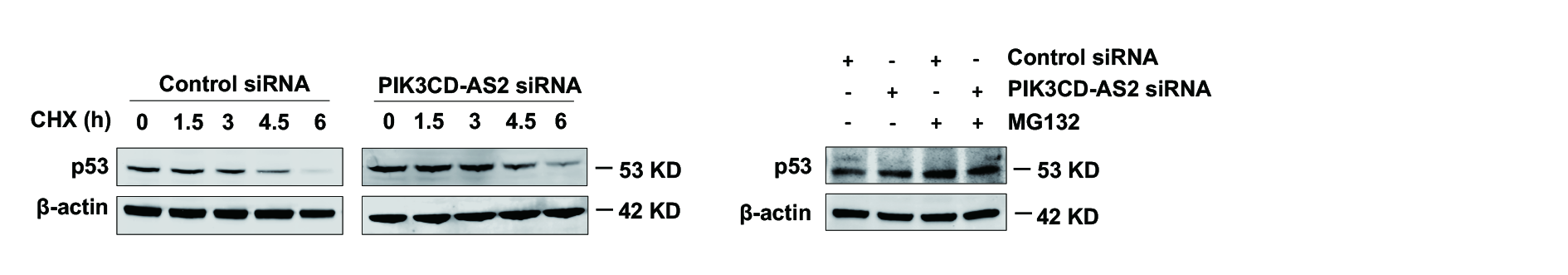

Supplement: Supplementary file 12 — Supplementary figure 8 [file 41389_2020_217_MOESM12_ESM.tif]
